# Computational genomics insights into cold acclimation in wheat

**DOI:** 10.3389/fgene.2022.1015673

**Published:** 2022-10-20

**Authors:** Youlian Pan, Yifeng Li, Ziying Liu, Jitao Zou, Qiang Li

**Affiliations:** ^1^ Digital Technologies, National Research Council Canada, Ottawa, ON, Canada; ^2^ Department of Computer Science, Department of Biological Science, Brock University, St. Catharines, ON, Canada; ^3^ Aquatic and Crop Research and Development, National Research Council Canada, Saskatoon, SK, Canada; ^4^ National Key Laboratory of Crop Genetic Improvement, Huazhong Agricultural University, Wuhan, Hubei, China

**Keywords:** cold acclimation, transcriptomics, lipidomics, phosphatidylglycerol lipid, differential expression feature extraction, RNA-seq, wheat

## Abstract

Development of cold acclimation in crops involves transcriptomic reprograming, metabolic shift, and physiological changes. Cold responses in transcriptome and lipid metabolism has been examined in separate studies for various crops. In this study, integrated computational approaches was employed to investigate the transcriptomics and lipidomics data associated with cold acclimation and vernalization in four wheat genotypes of distinct cold tolerance. Differential expression was investigated between cold treated and control samples and between the winter-habit and spring-habit wheat genotypes. Collectively, 12,676 differentially expressed genes (DEGs) were identified. Principal component analysis of these DEGs indicated that the first, second, and third principal components (PC1, PC2, and PC3) explained the variance in cold treatment, vernalization and cold hardiness, respectively. Differential expression feature extraction (DEFE) analysis revealed that the winter-habit wheat genotype Norstar had high number of unique DEGs (1884 up and 672 down) and 63 winter-habit genes, which were clearly distinctive from the 64 spring-habit genes based on PC1, PC2 and PC3. Correlation analysis revealed 64 cold hardy genes and 39 anti-hardy genes. Cold acclimation encompasses a wide spectrum of biological processes and the involved genes work cohesively as revealed through network propagation and collective association strength of local subnetworks. Integration of transcriptomics and lipidomics data revealed that the winter-habit genes, such as *COR413-TM1*, *CIPKs* and *MYB20*, together with the phosphatidylglycerol lipids, PG(34:3) and PG(36:6), played a pivotal role in cold acclimation and coordinated cohesively associated subnetworks to confer cold tolerance.

## Introduction

Wheat is the second most-produced cereal crop in the world; its yield and quality are severely affected by abiotic stress such as cold. During exposure to low but non-freezing temperature, plants increase their freezing tolerance in a process termed cold acclimation. Cold acclimation is a multi-genic processes, involves reprogramming of the transcriptome, proteome, lipidome, and metabolome, affects signaling between subcellular organelles, and induces significant changes in physiological processes and morphology (Li et al., 2018; Fürtauer et al., 2019; Li et al., 2021).

In response to cold stress, genetic and molecular analyses have identified dehydration-responsive element-binding protein 1/C-repeat binding factors (DREB1s/CBFs) as master transcription factors that regulate expression of cold regulated genes (CORs) during cold acclimation (Maruyama et al., 2009; Shi et al., 2018; Kidokoro et al., 2022). Many transcription factors regulate the cold-inducible expression of *DREB1* gene in the very complex manner (Thomashow, 2010; Kidokoro et al., 2020). In the downstream, *DREB1/CBF* transcription factors upregulate many cold-responsive genes (CORs). Multiple COR genes are identified as CBF regulon (Tchagang et al., 2017; Song Y. et al., 2021; Liu et al., 2021) with respect to multiple stresses such as cold, heat, drought, and salt. The expression of the COLD REGULATED 314 THYLAKOID MEMBRANE 1 (*COR413-TM1*) correlates with cold tolerance (Breton et al., 2003). Overexpression of *DREB1A* (*CBF3*) improves stress tolerance to both freezing and dehydration in transgenic plants. Under cold and dehydration conditions, the expression of many genes encoding starch-degrading enzymes changes dynamically; many monosaccharides, disaccharides, trisaccharides, and sugar alcohols accumulate in Arabidopsis (Maruyama et al., 2009).

Winter habit plants require prolonged exposure to cold, such as winter, to promote flowering in spring through a process known as vernalization (Chouard, 1960; Amasino, 2005; Kim et al., 2009). The two important evolutionarily adaptive mechanisms, cold acclimation for winter hardiness and vernalization, are thus initiated within the same time frame upon low temperature exposure (Limin and Fowler, 2002; Danyluk et al., 2003; Li et al., 2018). Studies in Arabidopsis have shown that epigenetic regulation of *FLC* (FLOWERING LOCUS C) plays an important role in the vernalization (Crevillen et al., 2014); whereas, the FLC genes in cereal plants appear to be implicated in many other aspects of plant growth and development in addition to vernalization (Kennedy and Geuten, 2020).

In wheat, *VRN1*, together with *VRN2* and *VRN3*, forms a pivotal regulatory module for its vernalization process (Oliver et al., 2009; Chen et al., 2018). Genetic studies revealed that the two loci on chromosome 5A, Frost Resistance-1 (*FR-1*) and *FR-2* affect freezing tolerance and winter hardiness of the temperate cereal plants (Knox et al., 2010; Fowler et al., 2016). *FR-1* is believed to be a pleiotropic effect of *VRN-A1* (Brule-Babel and Fowler, 1988). The *FR-2* QTL loci spanning on chromosome 5A contains a number of genes including a cluster of 21 genes encoding CBFs which are involved in cold acclimation (Vágújfalvi et al., 2003). *VRN-A1* appears to down-regulate the expression of COR genes in the CBF regulon adjacent to the *FR-2* locus in cold acclimated winter cereals (Limin and Fowler, 2006) indicating an interaction between *VRN-A1* and *FR-2* loci (Zhu et al., 2014). Low temperature induces the expression of *VRN1s*, while genes in cold pathways including CBFs and CORs are repressed (Danyluk et al., 2003; Li et al., 2018). On the other hand, CBF proteins are believed to directly bind the promoter of the *VRN1s* to repress flowering by negatively regulating its expression in cereals (Dhillon et al., 2010; Deng et al., 2015).

Changes in membrane fluidity, cytoskeleton rearrangement, and calcium influxes are among the earliest events taking place in plants upon exposure to low temperatures (Browse and Xin, 2001; Kidokoro et al., 2022). Membrane lipid unsaturation has been well documented for its role in low temperature adaptation in plants (Wolf et al., 2001; Zheng et al., 2021), while a lower membrane unsaturation level is favored under high temperature (Murakami et al., 2000). In addition, the level of desaturated Phosphatidylglycerol (PG) which contains a combination of 16:0, 18:0 and 16:1-*trans* fatty acids in PG is related to low temperature adaptability of plants (Murata and Los, 1997). Moreover, the reduction of *trans*-Δ3 hexadecenoic acid (t16:1) has been shown to be correlated with freezing tolerance, especially in cereal crops such as wheat (Huner et al., 1989; Li et al., 2021). Studies have also proposed that adjustment in lipid redistribution between the two glycerolipid pathways as well as lipid exchanges between the ER and chloroplast is critical for temperature adaptation in plants (Li et al., 2015).

Our previous study investigated the interactions between vernalization and cold acclimation pathways in the crown tissue (Li et al., 2018). Further analysis in leaf tissue revealed a mechanistic role of *trans*-16:1 in PG as a specific metabolite marker for screening freezing tolerance in wheat and genes in lipid pathways were specifically investigated (Li et al., 2021). However, the complexity of gene regulatory networks involved in mediating cold responses as well as lipid metabolism in leaves has not been fully explored. In this paper, we employed four wheat genotypes, winter habit Norstar (N), spring habit Manitou (M), and their near isogenic lines (NILs), winter Manitou (WM) and spring Norstar (SN) with the *VRN-A1* alleles swapped (Limin and Fowler, 2002), to study the cold acclimation process in leaves through computational pattern recognition, principal component analysis, and genes and lipids association networks. Genes associated with cold acclimation are identified and characterized.

## Results

### Transcriptome overview

**FIGURE 1:**
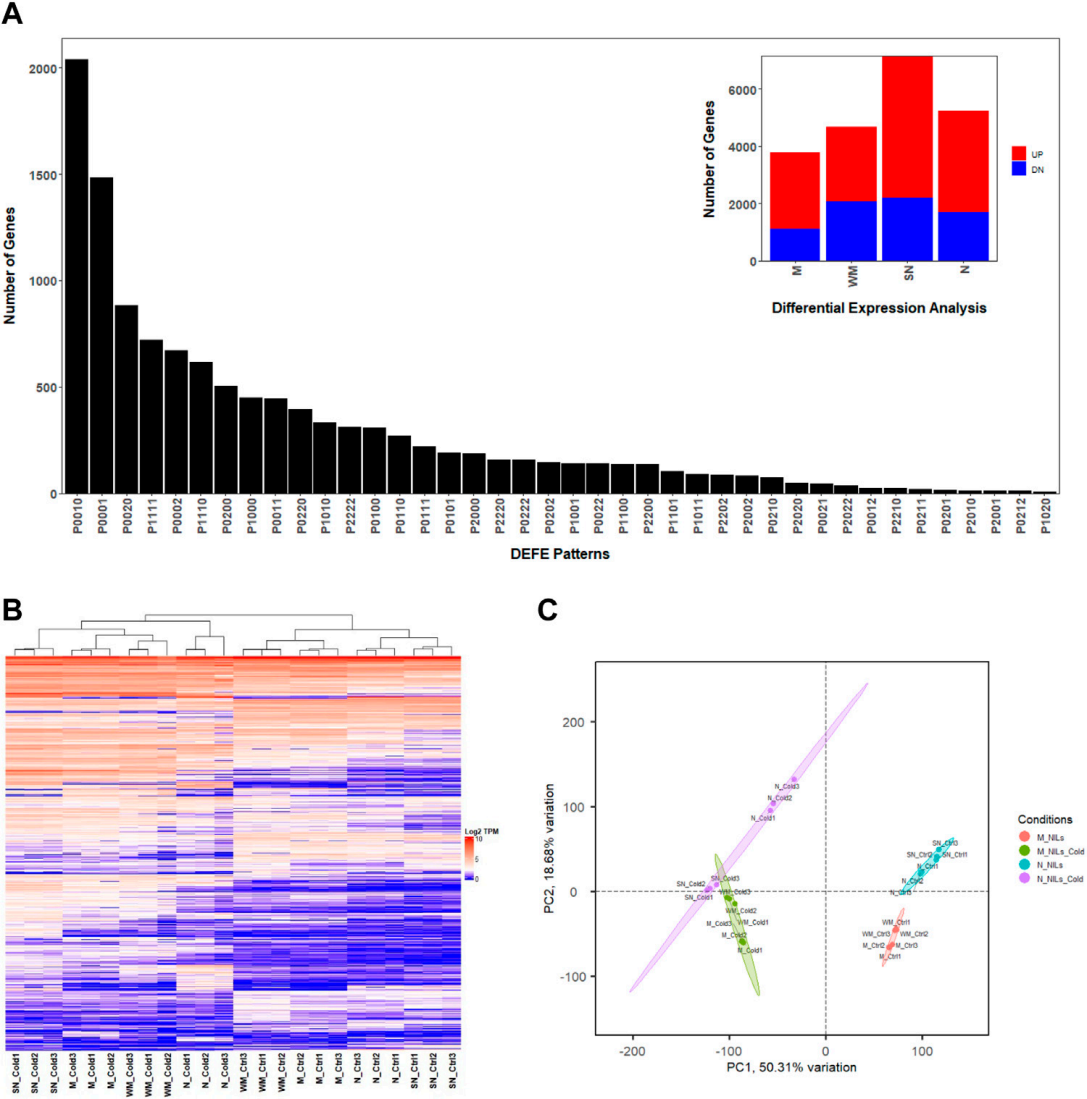
Transcriptome overview based on the 12,676 DEGs. **(A)** Frequency distribution (insert) and top 40 patterns of P series of DEFE analysis; **(B)** Heatmap; **(C)** Principal component analysis, where PC1 and PC2 are principal components 1 and 2, respectively.

In this study, RNA-seq data were obtained from leaves grown under low temperature treatment of four wheat genotypes with different LT50s (temperatures at which 50% of a population survives in an artificial freeze test), including Norstar (N, LT50 = −21.7°C), Manitou (M, LT50 = −8.3°C) and their near isogenic lines (NILs) spring Norstar (SN, LT50 = −13°C) and winter Manitou (WM, LT50 = −13.2°C) with the *VRN-A1* alleles swapped (Li et al., 2021). On average, 93% of the 26 million reads per sample that satisfied filtering criteria were mapped to the 106,914 known high confident wheat genes in the IWGSC RefSeq v2.1 genome assembly, from which, 12,676 differentially expressed genes (DEGs, Supplementary File S1) were identified based on criteria provided in the Materials and Method Section (|log2 fold-change| ≥ 2, adjusted *p*-value ≤ 0.01, and the maximum number of transcripts per million reads in a pair of compared samples ≥2, Figure 1). Between the cold treated samples and controls, the number of DEGs were spring Norstar (SN) > Norstar (N) > winter Manitou (WM) > Manitou (M) (Table 1). When the winter-habit genotype was compared with its respective near isogenic line (NIL) of spring-habit genotype, the difference between N and SN was more than three times as many DEGs as between WM and M in the cold treated samples, but the difference was only at about 1.4 times in the control samples (Table 1).

**TABLE 1:** Number of DEGs in each pair-wise comparison.

	Cold/Control				Cold treated		Control	
	M	WM	N	SN	WM/M	N/SN	WM/M	N/SN
Up	2633	2588	3522	4932	360	1515	522	856
Down	1127	2079	1702	2210	719	2085	407	431

Three series of differential expression feature extraction (DEFE, Pan et al., 2022) analyses were performed. The P series [the cold treated samples compared with their respective controls, P(M, WM, SN, N); *see* Material and Method Section for details] revealed the numbers of DEGs unique to each genotype were SN (2920, P0010 = 2036, P0020 = 884) > N (2156, P0001 = 1484, P0002 = 672) > WM (811, P0100 = 308, P0200 = 503) > M (637, P1000 = 450, P2000 = 187, Figure 1A). There were 1035 common DEGs (DEFE patterns: P1111 = 722, P2222 = 313) among the four wheat genotypes (Figure 1A). Across all four genotypes, there were more DEGs up-regulated than down-regulated when subjected to cold treatment (Table 1). The top DEFE expression pattern was unique up-regulation to SN (P0010 = 2036), which were followed by those unique up-regulation to N (P0001 = 1484). Among the number of uniquely down-regulated genes, SN had the highest number (P0020 = 884) and followed by N (P0002 = 672). Gene Ontology enrichment analyses of the genes unique to each genotype and common DEGs are available in Supplementary File S2.

Principal component analysis indicated that over 78% of variance were explained collectively by the first three principal components (PC1, PC2, and PC3). PC1 explained 50% variance related to cold treatment and clearly separated cold treated samples from the controls (Figure 1C and Supplementary Figure S1A). PC2 explains over 18% of variance in the differences between the two pairs NILs, and to some degree the between winter-habit and spring-habit as well (Figure 1C and Supplementary Figure S1B). PC3 explained 9% variance mainly associated with difference between winter-habit and spring-habit (Supplementary Figure S1).

Within each pair of NILs, we sought to understand the difference between genotypes of winter-habit (WM and N) and spring-habit (M and SN), and also the similarity and difference in gene expression profiles between the two pairs of NILs (N vs. SN; WM vs. M). Under cold treatment, we identified 1515 up-modulated genes and 2085 down-modulated between the winter-habit Norstar as compared to its spring-habit counterpart spring Norstar (C*1, C*2, Supplementary Figure S2A), the majority of which were unique to the N and SN pair (C01 = 1284, 85%; C02 = 1883, 90%). In comparison, the contrast between winter Manitou and Manitou was smaller (C1* = 360, C2* = 719; C10 = 171, 48%; C20 = 475, 66%). The disparity in the number of DEGs between these two pairs of NILs appeared to be related to the difference in freezing tolerance (delta LT50) between winter-habit and spring habit genotypes in each NIL. The delta LT50 is 8.7 between N and SN, but 4.9 between WM and M. Between the two pairs of NILs under cold treatment, they shared 189 genes up- and 202 down-modulated genes of winter-habit genotypes *versus* their respective spring-habit counterparts (C11, C22). In the control samples, the difference between the winter-habit and the spring-habit genotypes within each pair of NILs were not as drastic as those under cold treatment (Supplementary Figure S2B). Collectively, between the winter-habit and spring-habit genotypes, we found 4246 DEGs when treated with cold (C**), but 1898 DEGs in the controls (K**).

### Genes specific to winter-habit and spring habit

**FIGURE 2:**
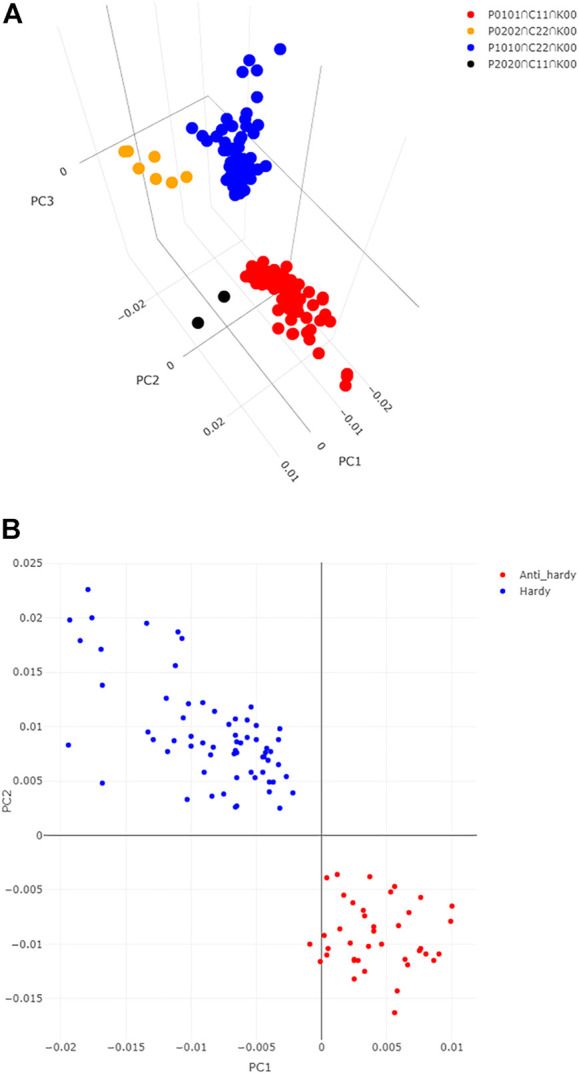
Distinction of genes associated with cold acclimation from the others. **(A)** Distinction of the 63 WHGs from the other three groups of DEGs identified in DEFE analysis as revealed by their scores of the first three principal components. **(B)** Distinction of the 64 cold hardy genes from the 39 anti-hardy genes revealed by their scores of the first two principal components. Where, PC1, PC2, and PC3 are the principal components 1, 2, and 3.

With regard to the winter-habit specific genes, we were particularly interested in those that were commonly differentially expressed in both winter-habit genotypes (N and WM), but not in either of the spring-habit genotypes (SN and M) when they were subjected to cold treatment. These genes could be represented by DEFE patterns P0101 (191 genes) and P0202 (144 genes) for up- and down-regulation, respectively. The genes up- or down-regulated in spring-habit, but not winter-habit genotypes as a result of cold treatment were represented by P1010 (335 genes) and P2020 (48 genes). Under cold treatment, up- or down-modulated in the winter-habit when compared with their spring-habit NIL pair (C11 = 189, C22 = 202), but not in the controls (K00), could be considered as supporting evidence of functional significance in low temperature adaptation. Integrating these three series of DEFE patterns, 63 genes were found to be up-regulated by cold, specific to both winter-habit genotypes (P0101∩C11∩K00 = 63, Table 2), while seven genes were down-regulated (P0202∩C22∩K00 = 7). On the contrary, 64 genes were found to be up-regulated by cold, specific to both spring-habit genotypes (P1010∩C22∩K00 = 64), while two genes were down-regulated (P2020∩C11∩K00 = 2) (*see* Supplementary File S3 tab Lists). These four groups of genes were distinctive in the three dimensional space represented by the first three principal components (PC1, PC2, and PC3 (Figure 2A and Supplementary Table S1).

**TABLE 2:** Winter-habit genes.

Gene_ID	Gene name	Gene description
TraesCS5B03G0571900		AAA-ATPase At5g57480
TraesCS4B03G0940000		acid phosphatase 1-like
TraesCS2D03G0746300		amino acid transporter AVT1I-like
TraesCS7A03G1216800		Basic helix-loop-helix dimerisation region bHLH domain containing protein
TraesCS4D03G0229100		Basic-leucine zipper (BZIP) transcription factor family protein
TraesCS3D03G0865700	BGLU42	Beta-glucosidase 42
TraesCS2D03G1058400	CHL	chloroplastic lipocalin-like
TraesCS1B03G1168700	COR413-TM1	Cold acclimation protein COR413-TM1, Cold-regulated 413 inner membrane protein 1, chloroplastic
TraesCS5A03G1113800		Cytochrome P450 family protein
TraesCS4D03G0748700	DEFL8	Defensin-like protein 1
TraesCS6D03G0772500	DHN3	dehydrin DHN4-like
TraesCS4D03G0668900		embryonic protein DC-8-like isoform X1
TraesCS2A03G0994800	ERF039	Ethylene-responsive transcription factor ERF039
TraesCS6D03G0772300		filaggrin-2-like
TraesCS6B03G0877600		galactan beta-1,4-galactosyltransferase GALS1-like
TraesCS2D03G1214900		geraniol 8-hydroxylase-like
TraesCS2A03G0593000		high mobility group nucleosome-binding domain-containing protein 5-like
TraesCS2D03G0347900		Hypothetical conserved gene
TraesCS7D03G0087400		late embryogenesis abundant protein 6-like
TraesCS4A03G0856100		leucine-rich repeat receptor-like protein kinase PEPR1
TraesCS3A03G0832100		Lipase, GDSL domain containing protein
TraesCS1D03G0421900		low-temperature-induced 65 kDa protein-like isoform X1
TraesCS7B03G0198800	LYP6	Lysin motif-containing protein, Pattern recognition receptor, Peptidoglycan and chitin perception in innate immunit
TraesCS1A03G0908000		non-specific lipid-transfer protein 2-like
TraesCS1B03G1066700		non-specific lipid-transfer protein 2-like
TraesCS3D03G0964600		Non-specific serine/threonine protein kinase
TraesCS3A03G1036100		Non-specific serine/threonine protein kinase
TraesCS5A03G0796200		noroxomaritidine synthase 2-like
TraesCS5A03G0796300		noroxomaritidine synthase 2-like
TraesCS5B03G0828400		noroxomaritidine synthase 2-like
TraesCS5B03G0828500		noroxomaritidine synthase 2-like
TraesCS5D03G0752600		noroxomaritidine synthase 2-like
TraesCS1D03G0066300	OEP161	Outer envelope pore protein 16-1, chloroplastic
TraesCS2A03G0069900		Pectinesterase inhibitor domain containing protein
TraesCS2B03G0102400		Pectinesterase inhibitor domain containing protein
TraesCS1B03G0841700		Phosphatidylethanolamine-binding protein PEBP domain containing protein
TraesCS5D03G1149800		phytosulfokine receptor 1-like
TraesCS4A03G0858900		phytosulfokine receptor 2
TraesCS5D03G1149600		phytosulfokine receptor 2-like
TraesCS7A03G0380300		probable apyrase 3
TraesCS5A03G1073800		probable lactoylglutathione lyase, chloroplastic
TraesCS2B03G0580500		Protein of unknown function DUF1218 family protein
TraesCS2D03G0389100	DHFR	putative anthocyanidin reductase
TraesCS7D03G0803900		Seed maturation protein domain containing protein
TraesCS3B03G1352700	GER8	Similar to Germin-like protein 1–3
TraesCS2D03G0826700		Similar to gibberellin receptor GID1L2
TraesCS1B03G0752200		Similar to Glutathione S-transferase GST 41 (EC 2.5.1.18)
TraesCS7D03G0446000		Similar to Pyruvate dehydrogenase E1 alpha subunit (EC 1.2.4.1)
TraesCS7A03G0517200		TB2/DP1 and HVA22 related protein family protein
TraesCS2B03G0488200	GL7	TON1 RECRUIT MOTIF (TRM)-containing protein, Regulation of grain size and shape
TraesCS2A03G0367000	GL7	TON1 RECRUIT MOTIF (TRM)-containing protein, Regulation of grain size and shape
TraesCS5A03G0532400	MYB20	Transcription factor MYB20
TraesCS4B03G0828000		uncharacterized protein LOC123093546 isoform X1
TraesCS5D03G0225200		uncharacterized protein LOC123124437
TraesCS7B03G0888400		uncharacterized protein LOC123162191
TraesCS7D03G0109900		uncharacterized protein LOC123168984
TraesCS2A03G0862400		Zinc finger, RING/FYVE/PHD-type domain containing protein
TraesCS2A03G1086200		
TraesCS3D03G0047400		
TraesCS4D03G0738600		
TraesCS5A03G0028100		
TraesCS5A03G0564900		
TraesCS5A03G1156200		

We named the 63 genes up-regulated specifically in both winter-habit genotypes (P0101∩C11∩K00) as ***winter-habit genes (WHGs)*** and the 64 genes up-regulated specifically in both spring-habit genotypes as ***spring-habit genes (SHGs)***. Gene Ontology enrichment analysis indicated that WHGs were highly represented by genes with functions in cold acclimation, embryo development ending in seed dormancy, regulation of monopolar cell growth, response to abscisic acid, response to lipid, oxidoreductase activity, acting on paired donors, with incorporation or reduction of molecular oxygen, and heme binding among others (*see* Supplementary File S3 tab GO_P0101∩C11∩K00). On the other hand, four of the seven genes suppressed by cold treatment in the two winter-habit genotypes had GO annotations that were enriched with calcium-dependent phospholipid binding (TraesCS1B03G0711800), passive transmembrane transporter activity (TraesCS5B03G0835100), fatty acid biosynthetic process (TraesCS7D03G0081000), and glucosidase activity (TraesCS7A03G0020800) that includes sucrose alpha-glucosidase activity (GO:0004575) and beta-fructofuranosidase activity (GO:0004564) (*see* Supplementary File S3 tab GO_P0202∩C22∩K00).

Under cold treatment, the 64 up-regulated genes specific in the two spring-habit genotypes (P1010∩C22∩K00) were enriched with phosphatidylethanolamine binding, photoperiodism, flowering, oxidoreductase activity, acting on single donors with incorporation of molecular oxygen, S-adenosylmethioninamine biosynthetic process, RNA polymerase II transcription regulatory region sequence-specific DNA binding, and inositol 3-alpha-galactosyltransferase activity among others (*see* Supplementary File S3 tab GO_P1010∩C22∩K00). Genes down-regulated in the spring-habit genotypes were enriched with cell redox homeostasis, and protein-disulfide reductase activity (*see* Supplementary File S3 tab GO_P2020∩C11∩K00).

**FIGURE 3:**
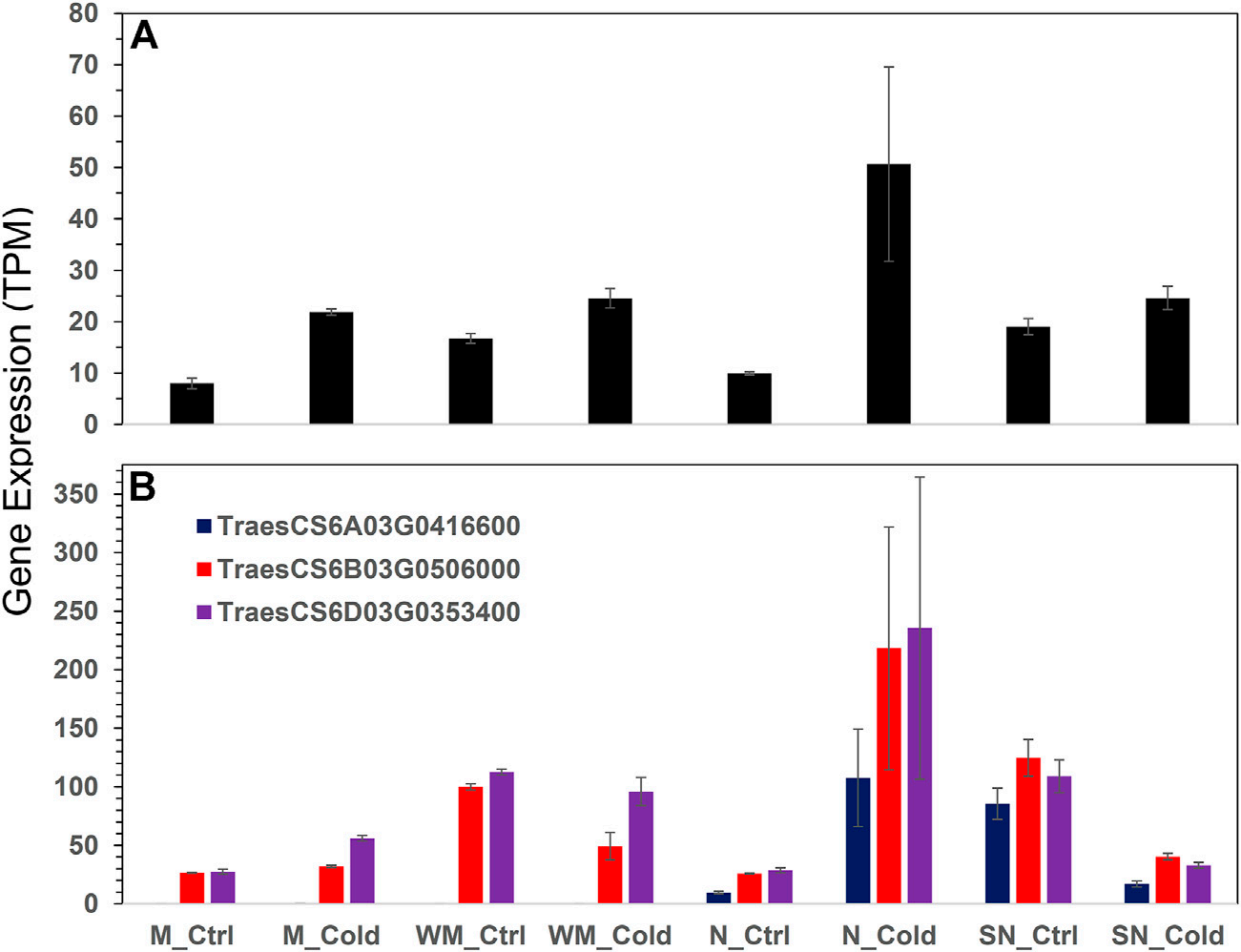
Expression of peroxiredoxins across all samples. **(A)** peroxiredoxin-2F, mitochondrial isoform X1, **(B)** peroxiredoxin-2E-2, chloroplastic-like. Error bars are one standard error of the mean of three replicates.

Both WHGs and SHGs groups were enriched with genes encoding oxidoreductase enzyme activities. In this regard, the 63 WHGs included genes encoding a geraniol 8-hydroxylase-like (TraesCS2D03G1214900), an indole-2-monooxygenase-like isoform X1 (TraesCS5A03G1113800), and five noroxomaritidine synthase 2-like (TraesCS5A03G0796200, TraesCS5D03G0752600, TraesCS5B03G0828500, TraesCS5A03G0796300, and TraesCS5B03G0828400); these seven genes acted on paired donors (GO:0016705). Whereas, the 64 SHGs include genes encoding a lipoxygenase (TraesCS5D03G0104900, EC:1.13.11.12), a linoleate 9S-lipoxygenase (TraesCS6B03G0405500, EC:1.13.11.58), and two uncharacterized proteins both involved in oxidoreductase activity and metal ion binding (TraesCS6D03G0269100 and TraesCS6D03G0269200); these four genes acted on single donors (GO:0016701). We thus further looked into the redox pathway and uncovered four peroxiredoxin genes for the DEG list (Figure 3). They were all highly expressed in the most winter hardy Norstar under cold treatment, but the three gene encoding peroxiredoxin-2E-2 were down-regulated in spring Norstar.

### Genes associated with cold hardiness

We scaled cold hardiness of each genotype based on their LT50 value (Li et al., 2021) according to the following formula (Table 3):

H=LT50/−25 (1)

where, H is termed as ***cold hardiness index***, LT50 is the half lethal temperature of a genotype, −25°C is the temperature below which most wheat genotypes would perish (Skinner and Garland-Campbell, 2008; Li et al., 2021). We considered a gene to be associated with cold hardiness, and therefore defined as ***cold hardy gene*** when the log2 fold change values and the expression values of the four genotypes under cold treatment were both significantly correlated (*p* ≤ 0.05) with the defined cold hardiness (Table 3); in addition, the expression values in all four genotypes under cold treatment were higher than their controls. Conversely, a gene would be considered ***anti-hardy*** when *1*) it was significantly down-regulated by cold in the extreme hardy genotype Norstar, and *2*) both log2FC and expression values were negatively correlated with the defined cold hardiness index among the four genotypes (Table 3). From the 12,676 DEG, 64 emerged as cold-hardy genes (Table 4) and the anti-hardy genes accounted 39 (Supplementary File S4; also in Supplementary File S1, “1” and “−1” in tab DEGs col AH). These two group of genes had distinctive variance distribution in the expression profiles as revealed in PC1 and PC2 two dimensional space (Figure 2B).

**TABLE 3:** The cold hardiness indices of the four genotypes in this study.

	M	WM	SN	N
Index	0.332	0.528	0.52	0.868

**TABLE 4:** Cold-Hardy genes.

Gene_ID	Gene name	Gene description
TraesCS5A03G1126300		actin-depolymerizing factor 3
TraesCS7A03G0546400		alpha-1,3-arabinosyltransferase XAT3-like
TraesCS5A03G0248600		auxin-binding protein 4
TraesCS5D03G0169500		auxin-responsive protein IAA31-like
TraesCS4A03G0266600	CK1	CBL-interacting protein kinase 31-like isoform X2
TraesCS1A03G0304900		Conserved hypothetical protein
TraesCS7B03G1059600		Conserved hypothetical protein
TraesCS1D03G0737600	Oc2*	cysteine proteinase inhibitor
TraesCS3D03G0413100		cysteine proteinase inhibitor 12-like
TraesCS1B03G0882400	Oc2*	cysteine proteinase inhibitor-like
TraesCS3B03G0987700	P5CS2	delta-1-pyrroline-5-carboxylate synthase 2-like
TraesCS4B03G1005300		DIBOA-glucoside dioxygenase BX6-like
TraesCS7B03G0073300		early nodulin-93-like
TraesCS7D03G0286800	RAP2-9	ethylene-responsive transcription factor RAP2-9-like
TraesCS6B03G0877600		galactan beta-1,4-galactosyltransferase GALS1-like
TraesCS4A03G0679200		glucan endo-1,3-beta-glucosidase 7-like
TraesCS1D03G0221500		glutathione S-transferase 4-like
TraesCS7D03G0069600		Glutathione transferase
TraesCS1B03G0654800	SGPP	haloacid dehalogenase-like hydrolase domain-containing protein Sgpp
TraesCS4A03G0180900		Harpin-induced 1 domain containing protein
TraesCS7A03G1041100	NRT2.4	High-affinity nitrate transporter, Nitrate transport, Auxin signalin
TraesCS2A03G0054400		HR-like lesion-inducer family protein
TraesCS2B03G0082600		HR-like lesion-inducer family protein
TraesCS2D03G0055600		HR-like lesion-inducer family protein
TraesCS2D03G0812700	HST	Shikimate O-hydroxycinnamoyltransferase
TraesCS2B03G0514500		interferon-related developmental regulator 2-like
TraesCS2D03G0404700		interferon-related developmental regulator 2-like
TraesCS5A03G0564300		low molecular mass early light-inducible protein HV90, chloroplastic-like
TraesCS5A03G0864400		low temperature-induced protein lt101.2-like
TraesCS7A03G0398900	LYP6	lysM domain-containing GPI-anchored protein LYP6-like
TraesCS7D03G0383800	LYP6	lysM domain-containing GPI-anchored protein LYP6-like
TraesCS5D03G1068400		multiple inositol polyphosphate phosphatase 1
TraesCS3D03G0949700	ONAC041	NAC domain-containing protein 83-like
TraesCS3B03G0141200		non-specific lipid-transfer protein 4.1-like
TraesCS5B03G1309300		Nucleotide-diphospho-sugar transferase domain containing protein
TraesCS5B03G1309400		Nucleotide-diphospho-sugar transferase domain containing protein
TraesCS2A03G1236100		pectinesterase inhibitor 8-like
TraesCS4A03G1138500		probable glutathione S-transferase GSTU6
TraesCS1D03G0686300		probable membrane-associated kinase regulator 4
TraesCS2B03G0690600		probable receptor-like protein kinase At1g49730 isoform X1
TraesCS4D03G0736900		probable serine/threonine-protein kinase At1g01540
TraesCS5A03G0968800		protein AE7-like 1
TraesCS2D03G0911500		protein RKD5-like
TraesCS5D03G0561000		putative ripening-related protein 2
TraesCS7B03G0271100		pyruvate dehydrogenase E1 component subunit alpha-2, mitochondrial-like
TraesCS7D03G0446000		pyruvate dehydrogenase E1 component subunit alpha-2, mitochondrial-like
TraesCS1D03G0944000		ras-related protein RABA1f-like
TraesCS1B03G0360800		RING finger and transmembrane domain-containing protein 2-like
TraesCS6D03G0772500	DHN3	Salt-induced YSK2 dehydrin 3
TraesCS2B03G1308500	SAPK1	Serine/threonine protein kinase, Hyperosmotic stress respons
TraesCS5B03G1367500		serine/threonine-protein phosphatase PP1-like
TraesCS4B03G0915000		subtilisin-like protease 4
TraesCS1B03G0874000		transcription factor GAMYB-like isoform X1
TraesCS5B03G0149900		tuliposide A-converting enzyme 2, chloroplastic-like
TraesCS2D03G0234900		UDP-glucuronic acid decarboxylase 4-like
TraesCS7A03G0941200		uncharacterized membrane protein At1g16860-like
TraesCS7B03G0786000		uncharacterized membrane protein At1g16860-like
TraesCS7D03G0906100		uncharacterized membrane protein At1g16860-like
TraesCS5D03G0230900		uncharacterized protein LOC123119728
TraesCS2A03G0325900		uncharacterized protein LOC123187869
TraesCS3B03G0014400		
TraesCS3D03G0047400		
TraesCS5A03G0434000		
TraesCS7A03G0474100		

The differences in cold hardiness index between N and SN (0.348, or delta LT50 = 8.7) was 1.8 time of that between winter Manitou and Manitou (0.196 or delta LT50 = 4.9). We compared the same difference in fold changes of differential expression between the two pairs of NILs and found that 29 of the 64 cold hardy genes had the same or larger extent of difference in their differential expression between the two pairs. In contrast, none in the 39 anti-cold-hardy genes had such an extent of difference. Orthology search against *Brachypodium distachyon*, *Oryza sativa*, and *Arabidopsis thaliana* indicate that the 64 cold hardy genes include genes encoding auxin responsive protein IAA31-like and auxin-binding protein 4; an early nodulin, *OSENOD93B*; high-affinity nitrate transporter, *NRT2.4* that involves both nitrate transport and auxin signalling; HR-like lesion-inducer family protein, lysin motif-containing protein, *LYP6*; no apical meristem (NAM) protein domain containing protein and others (Table 4 and Supplementary File S4).

There were four cold hardy genes (6.25%) among the 63 WHGs, and the percentage of total 64 cold hardy genes in the entire list of 12,676 DEGs was 0.50%. Therefore, the WHGs contained over 10 time enrichment of cold hardy genes as compared to the entire list of DEGs. These four genes included a galactan beta-1,4-galactosyltransferase (TraesCS6B03G0877600, EC:2.4.1.-), a salt-induced YSK2 dehydrin 3 (*DHN3*, TraesCS6D03G0772500), and a pyruvate dehydrogenase E1 component subunit alpha 2, mitochondrial isoform (TraesCS7D03G0446000, EC:1.2.4.1) and an unknown gene (TraesCS3D03G0047400). Gene ontology enrichment analysis indicated that the 64 cold hardy genes were enriched with cellular response to water deprivation and cold, chloroplast mRNA processing, kinase inhibitor activity, cysteine-type endopeptidase inhibitor activity, auxin binding among others detailed in Supplementary File S4 tab GO_Hardy.

### Gene-lipid association network analyses

Since membrane lipids are known to be altered in response to cold stress and in cold acclimation processes (Li et al., 2015; Li et al., 2021), we combined the 12,676 DEGs with 224 lipid traits in association network and clustering analyses to explore associations between transcriptome and lipidome.

Correlation analyses between all 12,676 DEGs and 224 lipid traits together with five experimental conditions (cold treatment, winter-habit, spring-habit, winter-habit genotypes treated with cold, spring-habit genotypes treated with cold) indicated that majority (58 and 62 genes, respectively) of the 63 WHGs and 64 SHGs were positively correlated with the respectively designated experimental conditions (Supplementary File S3 tab geneTraitCor_R). These two groups of genes correlate with distinctive lipidomics profiles (Table 5). For example, the WHGs were positively correlated with phosphatidylglycerol lipids PG(34:3), PG(34:2), and PG(36:6), most of monogalactosyldiacylglycerol (MGDG) and digalactosyldiacylglycerol (DGDG), total phosphatidylcholine (PC) and total phosphatidylethanolamine (PE), but negatively with PG(34:4), PG(34:1) and total PG. Whereas, high percentage the SHGs were significantly correlated with lysophosphatidylcholines (LPCs) and phosphatidylinositols (PIs).

**TABLE 5:** Number of winter-habit genes (left^a^) or spring-habit genes (right) correlated with respective lipid species.

Winter habit genes				Spring habit genes		
	Pos	neg			Pos	neg
DGDG(34:2)	0	25		PG(36:1)	17	0
DGDG(34:1)	0	13		LPG(18:2)	23	0
DGDG(36:2)	0	11		LPC(16:0)	53	0
DGDG(36:1)	0	12		LPC(18:3)	35	0
MGDG(34:1)	0	13		LPC(18:1)	9	0
PG(34:4)	0	6		LPC(20:1)	14	0
PG(34:3)	53	0		Total_LysoPC	38	0
PG(34:2)	49	0		PC(34:2)	0	20
PG(34:1)	0	15		PC(38:2)	0	34
PG(36:6)	51	0		PE(32:1)	47	0
Total_PG_All	0	50		PE(32:0)	45	0
PC(34:3)	42	0		PE(36:2)	10	0
PC(36:5)	9	0		PE(36:1)	28	0
PC(36:4)	49	0		PI(34:3)	28	0
PC(38:5)	14	0		PI(34:1)	46	0
PC(38:4)	36	0		PI(36:6)	49	0
PC(38:3)	51	0		PI(36:5)	38	0
PC(40:5)	15	0		PI(36:3)	43	0
PC(40:4)	19	0		PI(36:1)	36	0
PC(40:2)	6	0		Total_PI	27	0
Total_PC	40	0		PS(36:5)	10	0
PE(32:3)	38	0		DAG(16:0/16:0)	49	0
PE(36:6)	7	0		DAG(18:3/16:1)	0	41
PE(36:5)	9	0		TAG(50:4)_16:1_acyl_containing	53	0
PE(36:4)	37	0		TAG(52:7)_16:1_acyl_containing	44	0
PE(38:4)	20	0		TAG(52:6)_16:1_acyl_containing	45	0
PE(40:3)	39	0		TAG(52:5)_16:1_acyl_containing	27	0
PE(40:2)	41	0		Total_TAG_16:1_acyl_containing	39	0
PE(42:4)	32	0		TAG(52:8)_18:3_acyl_containing	21	0
PE(42:3)	23	0		TAG(52:6)_18:3_acyl_containing	17	0
PE(42:2)	8	0		TAG(52:5)_18:3_acyl_containing	21	0
Total_PE	7	0		TAG(52:4)_18:3_acyl_containing	44	0
PI(34:4)	0	12		MGDG(36:1)	24	0
PI(36:4)	0	15		LPC(18:0)	45	0
PS(34:3)	0	58		LPE(16:1)	17	0
PS(34:2)	0	16		LPC(16:1)	27	0
PS(36:6)	37	0		LPE(18:1)	28	0
PS(36:4)	0	40		TAG(48:1)_16:1_acyl_containing	9	0
PS(38:5)	0	50				
PS(38:2)	0	10				
PS(42:3)	38	0				
PS(42:2)	21	0				
Total_PS	0	13				
DAG(18:2/16:1)	0	38				
TAG(54:8)_18:3_acyl_containing	6	0				
TAG(54:7)_18:3_acyl_containing	6	0				
TAG(52:4)_18:2_acyl_containing	14	0				
TAG(52:3)_18:2_acyl_containing	12	0				
PS(40:1)	0	21				
DAG(18:2/16:3)	44	0				
DAG(18:1/16:3)	45	0				

^a^ This table contains the lipid species having correlation with more than five genes. Otherwise, all data are available in Supplementary File S3 Tab geneTraitCor_R.

By using topology overlap matrix in the WGCNA R package (Langfelder and Horvath, 2008), the network association degree and cluster membership of each gene were obtained and presented in Supplementary File S1. Correlation analyses were performed between each gene cluster and each lipid trait in addition to experimental design. Among the 50 clusters generated, six were significantly correlated (*p* < 0.05) with cold treatment to the two winter-habit genotypes, and together contained 71% of the WHGs (Supplementary File S1 tab ClusterTraitCor). The remaining 18 WHGs were in another large cluster less significant correlated with cold treatment to the two winter-habit genotypes (*p* = 0.067). Similarly, other eight clusters were significantly correlated to cold treatment to the two spring-habit genotypes and contained all 64 SHGs that were found through the aforementioned DEFE analysis. The cluster membership of these two groups was distinct. The association network analysis showed that none of the genes in the two groups were directly associated, through neither their immediate nor secondary neighboring nodes (Supplementary File S3 tabs WHGs_nodes and SHGs_nodes), thereby implying distinct functional space between the two groups of genes and lipids.

Similar analysis between the cold hardy genes and anti-hardy genes were conducted. These two groups were also well separated by distinctive lipidomic profiles, subnetworks, and cluster membership. Interestingly, all 64 cold hardy genes were positively collected with PG(34:3) and PG(36:6). In addition, the majority (64%) of the cold hardy genes were negatively correlated with PG(34:4) (Supplementary File S4 tab geneTraitCor_R).

As for network analysis, we extract the top 1% of the topology overlap matrix, which consists of 6,743 nodes connected with 825,776 edges. For the purpose of this study, we focus on the following subnetworks relevant to cold acclimation. We first defined the association strength (AS) of a subnetwork by using average connection degree of all nodes in the sub-network normalized by total number of nodes in the subnet:

$$AS=\frac{Average\;connection\;degree}{number\;of\;nodes\;in\;the\;subnet}$$ (2)

#### WHG subnet

**FIGURE 4:**
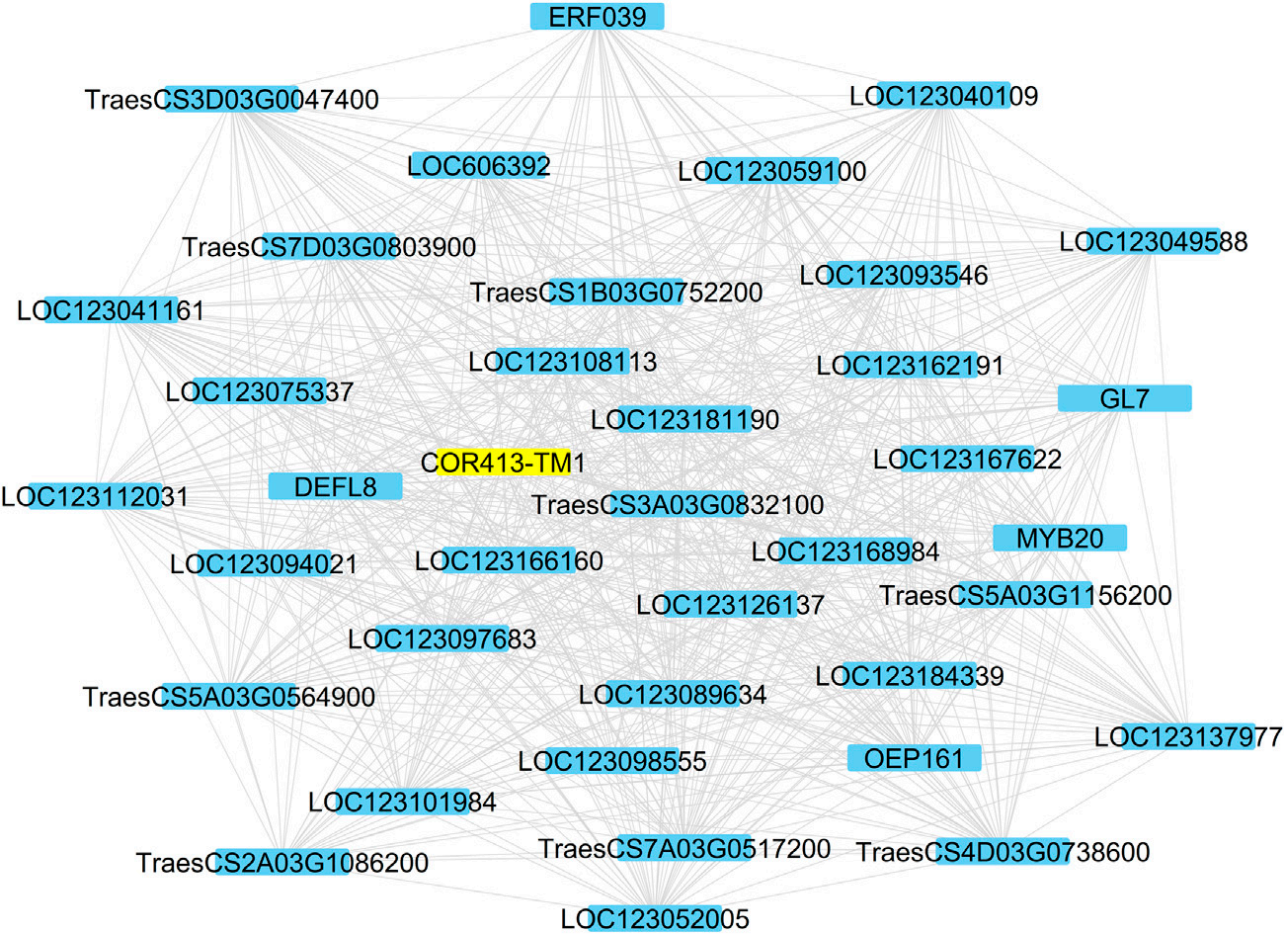
The cold acclimation genes directly associated with the Cold acclimation protein *COR413-TM1* highlighted. Details are available in Supplementary File S3.

**FIGURE 5:**
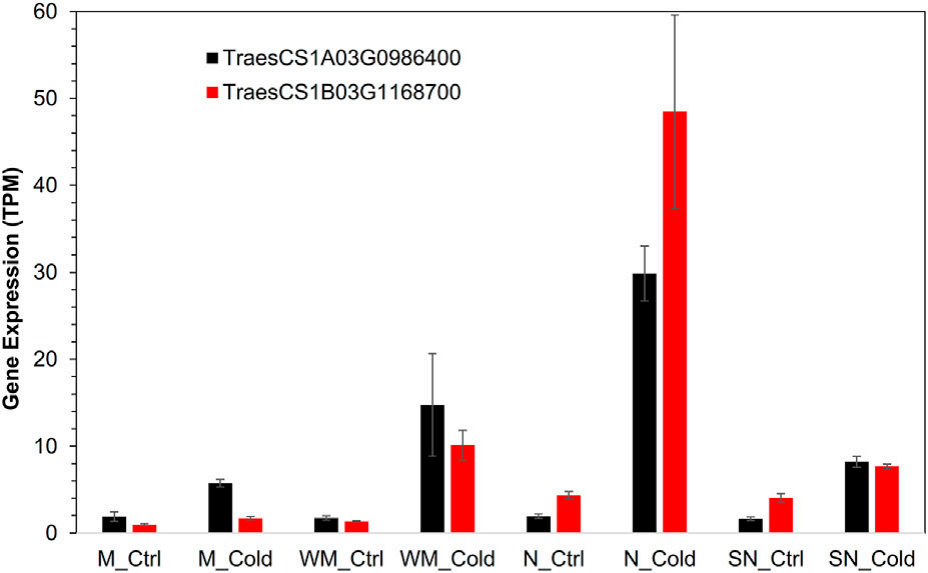
Expression of two homoeologs of *COR413-TM1* gene in different experimental conditions.

Fifty-one genes (81%) among the 63 WHGs were associated with at least one other gene in the group with an average connection degree of 33.5. Thus, the overall AS of the WHG subnet was 0.64. The top hub genes included a lipase, GDSL domain containing protein, orthologous to rice gene *OsGELP26* (Os01g0827700, connection degree = 44), a HVA22-like protein orthologous to *OsEnS-122* (Os08g0467500, 44), a galactan beta-1,4-galactosyltransferase (EC:2.4.1.-, 44), an AAA-ATPase orthologous to *At5g57480* (TraesCS5B03G0571900, 43), two pectinesterase inhibitor domain containing protein (Os04g0106000, 44 and 42), two non-specific serine/threonine protein kinases (TraesCS3D03G0964600, TraesCS3A03G1036100, both 42), a defensin (*DEFL8*, Os03g0130300, 42), pyruvate dehydrogenase E1 component subunit alpha (Os06g0246500, EC:1.2.4.1, 41), transcription factor *MYB20* (AT1G66230, 41), a Glutathione S-transferase *GST* (EC:2.5.1.18, 41), a pathogenesis-related transcriptional factor and ERF domain containing protein OsERF#034 (Os04g0550200, 40), and a cold acclimation protein *COR413-TM1* (Os05g0566800, 37). *COR413-TM1* was directly associated with all other hub genes mentioned above (Figure 4). A homoeolog of *COR413-TM1* on chromosome 1A was directly associated with PG(34:3). Both homoeologs of *COR413-TM1* were highly expressed in the two winter-habit genotypes (Figure 5). More details are available in Table 2 (Supplementary File S3 tab WHGs_nodes).

The associations among the 64 SHGs were very loose; only 27 genes (42%) have a direct neighbor within the group and spread over two subnets (Supplementary Figure S3). Collectively among the 27 genes, the association strength was 0.12 (Supplementary File S3 tab SHGs_nodes).

#### Cold hardy subnet

Similarly, we investigated the subnet of cold hardy *versus* anti-hardy genes. Among the 64 cold hardy genes, 61 (95%, Table 4 and Supplementary Figure S4A) were inter-associated with at least another gene within the subnet and have an association strength of 0.41. In addition, the cold hardy subnet had four points of contact with the WHGs subnet as described above. All the four points of contact were the hub nodes in both subnets. Phosphatidylglycerol lipids PG(34:3) and PG(36:6) were hub nodes in the cold hardy subnet with connection degrees of 22 and 10, respectively. Only 13 (33%) of the 39 anti-hardy genes have direct association with another gene within the group and they all were directly associated with PG(34:4) (Supplementary Figure S4B). More details are available in Supplementary File S4 tabs ColdHardy_nodes and AntiHardy_nodes). Two distinctive schools of network nodes were evident, one was represented by PG(34:3) and PG(36:6) and consisted of genes closely associated with cold hardiness, while the other was represented by PG(34:4) and consisted of genes closely associated with anti-hardy (Supplementary File S5).

#### Vernalization subnet

**FIGURE 6:**
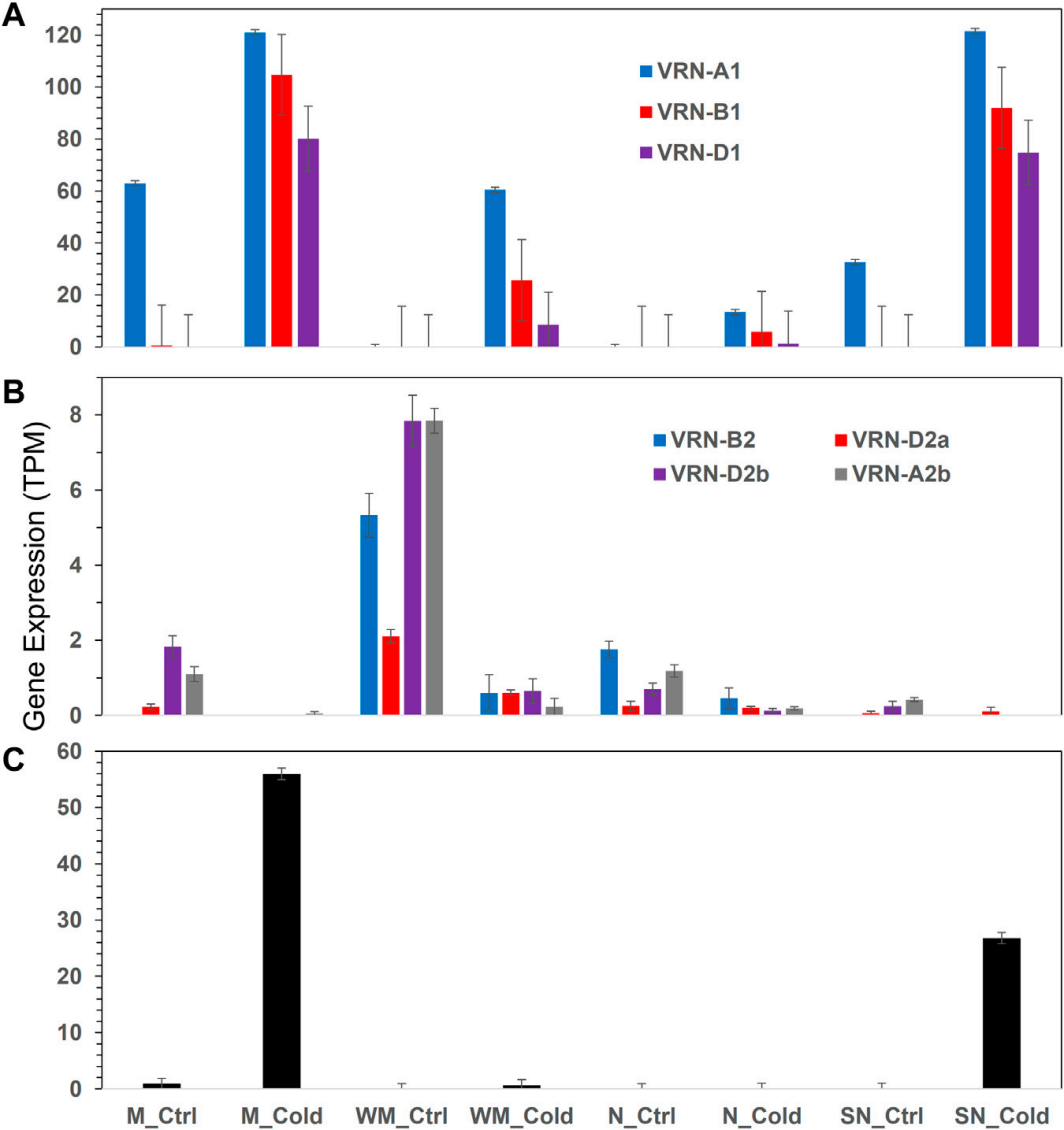
Expression of vernalization genes in different experimental conditions. **(A)** *VRN1*, **(B)** *VRN2*, **(C)** *VRN3*. Error bars are one standard error of the mean of three replicates. More details are available in Supplementary File S7.

The list of 12,676 DEGs included eight vernalization genes, three *VRN1* (TraesCS5A03G0935400, TraesCS5B03G0986000, and TraesCS5D03G0894800), four *VRN2* (TraesCS4B03G0958300, TraesCS4D03G0834500, TraesCS4D03G0834600, and TraesCS5A03G1265900), and one *VRN3* (TraesCS7B03G0031800) (Figure 6). The *VRN1* genes were highly up-regulated by cold in all four wheat genotypes, while their expression were higher in the two spring-habit genotypes (M and SN) than the winter-habit ones. The *VRN3* gene was up-regulated by cold treatment in the two spring-habit genotypes, while there was no effect in the two winter-habit genotypes. The *VRN2* were generally down-regulated by cold treatment. The *VRN2* and *VRN3* genes were not involved in the gene association network. Collectively, there were 214 genes in direct association with the three *VRN1*s, and they were interconnected to form a highly cohesive network with AS at 0.83. Nevertheless, these *VRN1* genes had no association even at the secondary neighborhood with either WHGs or cold hardy genes. From the 64 SHGs, one appeared in the *VRN1* immediate neighborhood and 42 other genes in the secondary neighborhood (Supplementary File S7). The *VRN-B1* (TraesCS5B03G0986000) had a direct association with the phospholipid-transporting ATPase (ALA1, EC:7.6.2.1, TraesCS4B03G0491700) as a single lipid gene in the immediate neighborhood of *VRN1* genes. There were other 19 lipid genes in the secondary neighborhood of *VRN1* genes.

## Discussions

### Overview–Integrative computational insights

Cold acclimation are investigated by integration of transcriptomics and lipidomics with various computational approaches including differential expression feature extraction, principal component analysis, correlation analysis, and gene-association network analyses. The differential expression feature extraction approach is a simple and effective pattern recognition method to find expression patterns in various conditions. Through integrating three differential expression feature extraction schemes, 63 winter-habit genes and 64 distinctive spring-habit genes are found. Correlation analysis reveals 64 cold hardy genes and 39 distinctive anti-hardy genes. The integration of transcriptomics and lipidomics analyses identifies two distinctive schools of network nodes (Supplementary File S5). The dimension reduction through principal component analysis is able to explain the majority of variance associated with cold treatment, cold hardiness, between winter-habit and spring-habit, and between two schools of network nodes in the reduced one, two, or three dimensional space represented by the first three principal components: PC1 for cold treatment, PC1+PC2 for cold hardiness and for two schools of network nodes, and PC1+PC2+PC3 for winter-habit and spring-habit. The distinction between the contrasting groups in each scenario is confirmed by integration of these methods. For example, the variance distribution with regard to the contrast between WHGs and SHGs is revealed by the differential expression feature extraction method and confirmed by principal component analysis, lipidomics association, and gene association network propagation. From gene association network perspective, the WHGs are highly associated among themselves as well as with others outside of the group. The association among the 64 SHGs, on the other hand, were very loose as indicated by the proportion of genes involved in the network and the network association strength. The same analogy is applied to the scenario between cold hardy and anti-hardy genes. These three scenarios of knowledge discovery indicate that there is no way of one-size-fit-all approach in the computational pipeline. Each case would have to be designed according to the characteristics of variance distribution in combination with domain knowledge. These pairwise contrasting analysis reveals that WHGs and cold hardiness are unique and yet they are inter-related to certain extent. They are two innate concerted efforts of plants to deal with cold stress.

### Limitations and complements

The four genotypes of reciprocal NILs used in this study inspire significantly to the design for this and earlier experiments and indeed help achieving much progress in the field of cold tolerance research in cereal plants (e.g. Limin and Fowler, 2002; Li et al., 2015, 2018, 2021). Nevertheless, the success of computational investigation requires significant sample size, balanced distribution of sample types, and data consistency within each type of samples. The most obvious limitation to the methods and analysis in this study is the small sample size of this dataset, which creates high imbalance between the sizes of sample space and the number of genes, known as the curse of dimensionality. Principle component analysis is a typical method for dimension reduction and able to explain the main variance in this study in one, two, or three dimensional spaces and reveals the distinction between contrasting groups.

The limitation of small sample size is most obvious in network analysis of this study, the size of eight samples is below the conventional necessity for a successful systemic network study such as the AraNet, which comprises from many distinct types of interactions, and millions of experimental or computational observations from diverse data types over decades of studies in *Arabidopsis thaliana* (Lee et al., 2010; Lee and Lee, 2017). To complement this limitation, we take the top 1% from the topology overlap matrix to reduce the false positive. Taking such high stringency would certainly sacrifice information. For example, the three *VRN1* homoeologs have similar network connections and high correlation in expression profile between themselves and in the same cluster; technically, they should be directly inter-connected too. But actually, they are not under the current selection criteria. The homoeologs of *COR413-TM1* are also in similar situation; the one in B sub-genome (TraesCS1B03G1168700) is not directly associated with PG(34:3), while the one in A sub-genome (TraesCS1A03G0986400) is. Therefore, caution should be taken at the interpretation of the result. This is complemented by network propagation in this study and such complement enables discovery of the fact that they share similar neighborhood nodes. Applying the small world social circle theory in humanity research, similar to the backbone theory used in WAGNA topology overlap matrix (Langfelder and Horvath, 2008), achieves the overall success of network analysis in this study. Such result is further strengthened through integration of strengths of other methods applied.

The prime condition in this study is cold treatment, which is reflected in two contrasting pairs (WHGs *versus* SHGs and cold hardy *versus* anti-hardy) which are revealed by one computational method and supported by PCA and at least one more other method in this study. The cohesiveness of group membership is reflected by an association strength in the membership in each of these groups and is contributed by environmental, physiological, and/or genetic factors. Both groups of WHGs and cold hardy genes have higher membership involvement and association strengths than their respective counterparts. Finally, the vernalization subnetwork is highly relevant to the genetic factor contributed by the vernalization through the recessive allele *vrn1* associated with *VRN-A1* locus. The association strength of vernalization subnetwork is 0.83, i.e., each gene is directly associated with 83% in immediate neighborhood of *VRN1*, which indicates the extent of contribution by genetic contribution to over winter cold/freezing tolerance of wheat plants.

### Insights into cold acclimation

Cold acclimation is a complex system and the 63 WHGs encompass a wide spectrum of biological processes. Gene association network analysis of WHGs subnet reveals that many hub genes in this group are directly associated collectively with over 70% of the genes in the group with an overall association strength of 0.64. This indicates that these genes coordinate in concerted manner to confer the common goal of cold tolerance.

#### Signaling of cold stress

In plants, the calcineurin B-like protein (CBL) family represents a group of calcium sensors and plays a pivotal role in decoding calcium transients by specifically interacting with and regulating a family of protein kinases (*CIPKs*). *CIPKs* is known to confer cold stress tolerance in cold acclimated *Arabidopsis thaliana* (Aslam et al., 2022), pepper, and tomato (Ma et al., 2022). Two CIPKs are among the hub genes of WHGs subnet in this study (connection degree = 42) and they are highly expressed in both winter-habit genotypes (WM and N) when treated with cold, but barely any expression in all other samples. They are directly associate with all hub genes in the subnet.

#### Involvement of carbohydrate metabolism in cold acclimation

Beta-glucosidases are the enzymes that catalyze the hydrolysis of terminal, non-reducing β-D-glucosyl residues from a variety of glucoconjugates which include glucosides, oligosaccharides, and 1-O-glucosyl esters (Godse et al., 2021). Beta-glucosidase is a rate-limiting enzyme that is involved in the hydrolysis of cellulose, affects cell wall structure, and plays a key role in cell adaptations to the physical deformations caused by cold stress (Sun et al., 2021). Beta-glucosidases hydrolyze inert precursors to release antioxidant substances under various abiotic stresses in rice (Opassiri et al., 2007). Expression of beta-glucosidase gene is induced in response to low temperature in chickpea (Khazaei et al., 2015). After cold acclimation, beta-glucosidase is require for freezing tolerance in *Arabidopsis thaliana* (Thorlby, 2004). In this study, the expression of the beta-glucosidase gene is upregulated to different extent in all four genotypes under cold stress and is much higher (>3 times) in both winter-habit genotypes than in the spring-habit genotypes.

#### Integrity of plasma membrane

Expression of lipocalins and lipocalin-like proteins in wheat (*Triticum aestivum*) is known to be associated with the plant’s capacity to develop freezing tolerance, cold acclimation induces a high accumulation of temperature-induced lipocalin TaTIL-1 in an enriched plasma membrane fraction of cold-acclimated wheat but not in nuclei (Charron et al., 2005). The chloroplastic lipocalin AtCHL is known to prevent lipid peroxidation and protect Arabidopsis against oxidative stress (Levesque-Tremblay et al., 2009) and is required for sustained photoprotective energy dissipation (Malnoë et al., 2018). In this study, the chloroplastic lipocalin-like gene (CHL) is highly up-regulated by cold in both winter-habit genotypes, but not (or a minor extent of down-regulation) in the two spring-habit genotypes.

#### Resistance to oxidative stress and cellular detoxification

Plant adaptation to low temperature not only induces lipid desaturation in cellular membranes but also generation of reactive oxygen species (ROS) and changes in redox state (Murata and Los, 1997; Wallis and Browse, 2002). The multifunctional enzymes glutathione S-transferases (GSTs) participate in oxidative stress resistance and cellular detoxification and highly associated with cold stress of Hami melon (Song W. et al., 2021) and pumpkin (Abdul Kayum et al., 2018). There are 92 *GSTs* or *GSTs* like in the DEGs list, the majority of them, including a key hub gene in the WHGs subnetwork (TraesCS1B03G0752200), are significantly upregulated by cold, especially in Norstar and spring Norstar.

The pyruvate dehydrogenase E1 component subunit alpha-2, mitochondrial isoform (*PDH-E1a*, TraesCS7D03G0446000, EC:1.2.4.1) appears to be a key hub gene in both WHGs and cold hardy subnets of this study. It’s homoeolog in chromosome B (TraesCS7B03G0271100) is also a cold hardy gene and up regulated by cold in all four genotypes. The pyruvate dehydrogenase (*PDH*) complex catalyzes the oxidative decarboxylation of pyruvate with the formation of acetyl-CoA, CO2 and NADH. Much of the studies were done with animal in relation to the effect of cold. For example, *PDH* is associated with metabolic rate depression during freezing and anoxia of wood frogs (Al-Attar et al., 2019) and during hibernation of ground squirrel (Herinckx et al., 2017). In plants, *PDH* is found in both chloroplast and mitochondria. The two genes described above encoding mitochondrial isoform in this study is truly up-regulated by cold, related to respiration and anoxia. Whereas, the chloroplast isoform concerns fatty acid synthesis (Li et al., 2021) and photorespiration (Blume et al., 2013). There are three homoeolog genes in this study (TraesCS2A03G0021400, TraesCS2B03G0027300, and TraesCS2D03G0019300) encoding pyruvate dehydrogenase E1 component subunit alpha 3, the chloroplastic isoform (*PDHA1*); they are all down-regulated by cold in all four wheat genotypes (Supplementary File S1).

Upon the cold treatment, the WHGs and SHGs show oxidoreductase activity with incorporation of molecular oxygen. But WHGs act on paired donors (EC1.14.-.-) and are from a family of heme-binding and iron containing enzymes. They catalyze an oxidation-reduction (redox) reaction in which hydrogen or electrons are transferred from reduced flavin or flavoprotein and one other donor; one atom of oxygen is incorporated into one of the donors. This group consists of seven genes including one encoding geraniol 8-hydroxylase-like, an indole-2-monooxygenase-like isoform X1, and five noroxomaritidine synthase 2-like. Their expressions are significant in the two winter-habit genotypes but not otherwise. Whereas, as represented by two lipoxygenases (EC1.13.11.-), SHGs act on single donor (EC1.13.-.-) are from a family of non-heme iron containing enzymes, mostly catalyze the dioxygenation of polyunsaturated fatty acids. It has been shown that low temperature or cold stress induced reactive oxygen species (ROS) production is often accompanied by lipid peroxidation and oxidative damage to cellular membranes (Kim et al., 2013).

A recent study showed that a thylakoid-associated protein, peroxiredoxin Q, is required for the production of t16:1 in chloroplast and photosynthesis systems (Lamkemeyer et al., 2006; Horn et al., 2020), indicating a link between t16:1 production and redox status. Three genes encoding the chloroplast peroxiredoxin-2E-2 were uniquely induced in Norstar (Figure 3) which might be related to the reduction in t16:1 levels. Also, the relationship between up-regulation of heme-binding proteins and stress tolerance in general, and specific with regard to cold tolerance. In *Arabidopsis thaliana*, the heme-associated protein *AtHAP5A* enhances freezing stress resistance and has significant effects on inhibiting cold-induced ROS accumulation and activating ABA-related genes’ expression (Shi et al., 2014).

#### Transcriptome regulation

Myeloblastosis transcription factors *MYB20* is a key hub gene in the WHGs subnet directly associated with 41 other genes. In *Arabidopsis thaliana*, *MYB20* is well known to acts as a negative regulator of plant response to desiccation and cold stress and its expression is reduced to less than half (Gao et al., 2014). Another study shows transgenic plants overexpressing *AtMYB20* (AtMYB20-OX) enhance salt stress tolerance while repression lines (AtMYB20-SRDX) are more vulnerable to NaCl than wild-type plants (Cui et al., 2013). The expression level of *MYB20* in this study is near 100 folds in the two winter-habit genotypes (WM and N) as compared to respective controls, and also over 10 folds as compared to the two spring-habit genotypes under cold treatment. *MYB20* is involved in the transcriptional network regulating the secondary wall biosynthetic program (Zhong et al., 2008). In addition, MYB proteins activate transcriptional repressors that specifically inhibit flavonoid biosynthesis, which competes with lignin biosynthesis for the aromatic amino acid phenylalanine precursors (Geng et al., 2020)

The COLD REGULATED 314 THYLAKOID MEMBRANE 1 (*COR413-TM1*) is an integral component of chloroplast inner membrane and well-known in cellular responses of plant to cold, water deprivation, cold acclimation and abscisic acid. *COR413-TM1* is characterized to provide normal freezing tolerance in *Arabidopsis thaliana* (Okawa et al., 2008), *Brachypodium distachyon* (Colton-Gagnon et al., 2014) and wheat (Breton et al., 2003). There are two homoeologs of *COR413-TM1* gene among the 12,676 DEGs in this study. The one on A sub-genome (TraesCS1A03G0986400) is a member of School B and a key hub gene in the PG(34:3) subnet (Supplementary File S6). The other on B sub-genome (TraesCS1B03G1168700) is a major hub gene in WHGs subnet and directly associated to all major hub genes in the subnet. Both *COR413-TM1* homoeologs are associated with over 400 DEGs and lipid species in this study.

### Vernalization, cold hardiness *etc.*

During the crossing process of the two wheat cultivars, the non-hardy spring wheat Manitou gained the *vrn-A1* loci and became winter Manitou, while the very cold hardy winter-habit Norstar, gained the dominant *Vrn-A1* locus and became spring Norstar. It is interesting to note that the LT50 value of the two NILs (WM and SN) are very close, but the change in LT50 is very different between the two pairs. Spring Norstar has a higher change (reduced by 8.7°C) in LT50 from Norstar, also has a highest number of DEGs as well as unique DEGs to the genotype when subjected to cold treatment. As a contrast, winter Manitou has a lower change (increased by 4.9°C) in LT50 from Manitou, also has a lower number of DEGs as well as unique DEGs to the genotype.

Cold acclimation and vernalization are two major mechanisms for winter survival in wheat (Li et al., 2018). Consistent with previous study on crown tissue, the *VRN1* genes including *VRN-A1*, *VRN-B1* and *VRN-D1* are induced at higher levels after cold treatment in Manitou and spring Norstar than that of in Norstar and winter Manitou. Vernalization requirement duration in winter wheat is controlled by *VRN-A1* at the protein level (Li et al., 2013). This is apparently relevant to the genetic background of vernalization genes in leaf and crown tissues as determined by the dominant allele *Vrn-A1* in Manitou and spring Norstar *versus* the recessive allele *vrn-A1* in winter Manitou and Norstar. As a result, integration of DEFE, PCA, gene association network analysis, and lipidomics analysis as discussed above, the 63 WHGs significantly expressed in winter-habit genotypes, winter Manitou and Norstar, are highly distinctive from the 64 SHGs. Such distinction is no doubt relevant to the genetic background of the four wheat genotypes.

The *VRN2* genes are known to be repressed by cold in cereal plants and the expression *VRN3* is subjected to negatively regulation by *VRN2* (Kim et al., 2009). Thus the down-regulation of *VRN2* in the leaf of this study permits the transient expression of *VRN3* gene. Also, the *VRN1* gene in cereals is known to plays a dual role of both a promoter of *VRN3* and a cold-activated repressor of *VRN2* (Kim et al., 2009). Our result is consistent in this regard.

### Concluding remarks

Cold acclimation and vernalization are major strategies for winter survival in wheat. The differential expression feature extraction enables the discovery of a group of 63 WHGs that are significantly expressed in both vernalized winter-habit winter Manitou and Norstar, but not in either Manitou or spring Norstar. These genes are cohesively associated with one another in their local subnetwork and have a distinctive lipidomics association to achieve survival in the cold stress. They encompass a wide spectrum of transcriptional reprograming that involves signaling, maintenance of plasma membrane fluidity and rigidity, cell energy and redox homeostasis, and transcriptional regulation. The phosphatidylglycerol lipids, PG(34:3) and PG(36:6), appear to be well associated with majority of these WHGs including *COR413-TM1*, which play an integral role in chloroplast inner membrane and the well-known in cellular responses of plant to cold, water deprivation, cold acclimation and abscisic acid. The PG(34:3) and PG(36:6) play a master role in cold hardiness. The discovered WHGs and cold hardy genes are highly distinctive as confirmed by PCA, network propagation, and/or lipidomics profiles. The three *VRN1* genes are closely associated with their immediate neighborhood, which are highly cohesive.

## Materials and methods

### Plant materials

Plant materials as detailed in Limin and Fowler (2002) include four wheat (*Triticum aestivum* L.) genotypes (M: Manitou, WM: winter Manitou, N: Norstar and SN: spring Norstar). Briefly, a non-hardy spring wheat Manitou, determined by dominant *Vrn-A1* allele, and a very cold hardy winter-habit Norstar, determined by recessive *vrn-A1* allele, were crossed to produce the reciprocal near-isogenic lines (NILs) (Limin and Fowler, 2002). During the crossing process, the vernalization allele in Norstar (*vrn-A1*) was replaced by the spring-habit allele at *Vrn-A1* locus from Manitou to produce spring Norstar. Whereas, replacing the *Vrn-A1* allele of Manitou with the *vrn-A1* from Norstar made Manitou a vernalization-responsive winter-habit genotype (winter Manitou).

Briefly, for cold treatment under controlled environments, wheat plants were grown in chambers with 16-h-light (∼120 μmol m^−2^ s^−1^) and 8-h-dark at 23°C up to the stage of four leaves (3 weeks) and then transferred to 4°C chamber for 6 weeks. The third fully opened leaves from cold-treated and untreated plants were collected at around 10:00 a.m. and immediately frozen in liquid N_2_. Samples were stored at −80°C until lipidomics and RNA-seq analyses. Each genotype under a condition has three independent biological replicates.

### RNA sequencing and data quality control and mapping

The RNA-seq dataset in Li et al. (2021) was reanalyzed in this study. Briefly, total RNA was extracted from 0.1 g wheat leaf tissues for each of the 24 cold treated and un-treated samples using the Agilent Plant RNA isolation kit (Agilent Technologies) and sequenced as described in Li et al. (2021). In total, the RNA-seq dataset contains 24 wheat samples with an average of 34 million reads per sample and available at Gene Expression Omnibus (GEO, GSE156300). We trimmed adaptor sequence, discarded low-quality reads (Phred Score ≤20) and eliminated short reads (length ≤20 bps) using a software package FASTX toolkit (http://hannonlab.cshl.edu/fastx_toolkit/). In average, 26 million reads remained and were aligned to the high confidence gene models in the IWGSC RefSeq Version 2.1 reference genome (Zhu et al., 2021) by using STAR (v2.7.10a, Dobin et al., 2013). From the BAM files generated by STAR, level of mRNA in each sample was quantified as transcript per million (TPM) by using RSEM (Li and Dewey, 2011).

### DEG analysis

Recent studies in RNA-seq data analysis indicate that normalized expression data, such as TPM, FPKM or RPKM is not acceptable for DEG analysis (Zhao et al., 2020; Zhao et al., 2021). The read count data from STAR above were used to perform eight pairwise gene differential expression analyses using DESeq2 (Love et al., 2014). Each of the four genotypes were compared between cold-treated and un-treated control (WMC-WMK, MC-MK, NC-NK, and SNC-SNK, where, W = winter, S = spring, M = Manitou, N=Norstar, C = cold, K = control). Similarly, within each pair of NILs (winter Manitou and Manitou, Norstar and spring Norstar), we compared winter-habit genotype with its spring-habit counterpart in the cold treated samples (WMC-MC, NC-SNC) as well as in controls (WMK-MK, NK-SNK). The outputs from DESeq2 include log2 fold change values and associated statistical significance (*p*-values, and adjusted *p*-values).

### Data reduction and partitioning

We applied the criteria of |log2FC| ≥ 2, adjusted *p* ≤ 0.01 and the max(TPM of compared samples) ≥ 2 to identify differentially expressed genes (DEGs). Differential Expression Feature Extract (DEFE) method (Pan et al., 2022) was applied to partition the DEGs into groups of various expression profiles, whether they were consistent across all genotypes in response to cold treatment or specific to each or certain pairs of genotypes. Three series of DEFE analyses were performed. Firstly, for the comparison between cold treated samples in the four genotypes *versus* their respective controls, a series of DEFE patterns were identified with a prefix “P” and followed by four digits each representing a genotype in the order of M, WM, SN, and N. Among the four digits, “0” means not differentially expressed, “1” denotes up regulated and “2” down-regulated. For example, P0210 represents a group of genes that were not differentially expressed in Manitou and Norstar, down-regulated in winter Manitou and up-regulated in spring Norstar when treated with cold. Similarly, we were able to obtain groups which were either consistent between the two winter-habit genotypes in response to cold treatment as well as in control, or they were specific to one individual NIL. The pattern ID in these two series start with either “C” or “K” for cold treated or control samples, respectively, and followed by two digits, representing WM/M and N/SN, respectively.

### Clustering, correlation, and gene association networks analyses

The WGCNA R package (Langfelder and Horvath, 2008) was used to cluster the normalized expression data of DEGs together with lipid traits based on the distance measure by topology overlap matrix (TOM). Hierarchical clustering was employed based on the similarity matrix to cluster genes as described in Pan et al. (2018). Briefly, the network connection weight was calculated based on TOM and the top 1% weight was used for network construction. The trait-trait, gene-trait, and cluster-trait correlation matrices were computed. Here, a trait refers to an experimental condition and a lipid species. Network visualization was performed by using Cytoscape (Shannon et al., 2003).

### Gene orthologue, annotation and GO enrichment analysis

For the known IWGSC RefSeq 2.1 genes, we obtained their orthologues in *Arabidopsis thaliana*, *Brachypodium distachyon*, *Oryza sativa* Japonica, and gene names and descriptions from EnsemblPlants (http://plants.ensembl.org/Triticum_aestivum/Info/Index) through reciprocal best kit BlastP (e ≤ 10^−5^). The orthologues, annotations, cluster membership, and mapping of gene IDs with various previous genome assembly are available in the Supplementary File S1.

GOAL software (Tchagang et al., 2010) was used in the gene ontology (GO) enrichment analysis. The GO terms were recently updated from EnsemblPlants release 51 (http://plants.ensembl.org/Triticum_aestivum/Info/Index) and the gene-GO association file for this version of wheat genome are available in the Supplementary File S8. An updated version of GOAL software is available at https://github.com/DT-NRC/GOAL2.0.

### Principal component analysis and visualization of data

Principal component analysis was performed by using PCAtools R package in Bioconductor (Blighe and Lun, 2022). The 12,676 DEGs were visualized in heatmap by using ComplexHeatmap R package in Bioconductor (Gu et al., 2016). Otherwise, R versions 4.2.1 were used in this study.

## Data Availability

The datasets presented in this study can be found in online repositories. The names of the repository/repositories and accession number(s) can be found in the article/Supplementary Material.

## Supplementary material

SUPPLEMENTARY FILE S1 Details of the 12676 differential expressed genes (DEGs) including their annotation, mapping of gene IDs to various previous genome versions, membership in various groups identified in this study. This data file contains three tabs: 1) DEGs: A list of 12676 DEGs, their annotation, orthologues, expression, DEFE pattern and their membership to various groups proposed; 2) DEFE Stats: Frequency statistics of all DEFE patterns; 3) ClusterTraitCor: Correlation between each of the 50 clusters with an experimental condition or a lipid species.

SUPPLEMENTARY FILE S2 Gene ontology (Biological Process) enrichment analyses for the genes uniquely up or down regulated by cold in one or all genotypes. This file contains 11 tabs leading by a “Summary” tab providing the statistics overview of the 10 groups of genes uniquely or commonly up or down regulated by cold in the four wheat genotypes. The subsequent tabs labelled by respective DEFE pattern ID followed by “BP” to provide details.

SUPPLEMENTARY FILE S3 Details of the group of 63 winter-habit genes (WHGs), spring-habit gene (SHGs) and others two groups presented in Figure 2A. This file contains 10 tabs: 1) Lists: membership in the four groups, their annotation, DEFE pattern, and the difference of fold changes between the two pairs of NILs (N/SN – WM/M); 2) geneTraitCor_R: Correlation coefficient of each gene with experimental conditions and with each lipid species; 3) - 6) Gene ontology enrichment analyses of the four groups labelled by their respective DEFE patterns: GO_P0101∩C11∩K00, GO_P0202∩C22∩K00, GO_P1010∩C22∩K00, GO_P2020∩C11∩K00; 7) - 10) Gene associate network analysis, nodes and edges of WHGs (P0101∩C11∩K00) and SHGs (P1010∩C22∩K00).

SUPPLEMENTARY FILE S4 Details of cold hardy genes and anti-hardy genes presented in Figure 2B. This file contains 8 tabs: 1) Lists: members in the two groups and their annotation; 2) geneTraitCor_R: Correlation coefficient of each gene with experimental conditions and with each lipid species; 3) - 4) Gene ontology enrichment analyses of the two groups labelled by their respective group names; 5) - 8) Gene associate network analysis of these two groups of genes including notes and edges.

SUPPLEMENTARY FILE S5 Description with supporting figures of the two schools of network nodes represented by phosphatidylglycerol lipids, PG(34:4) for School A, PG(34:3) and PG(36:6) for School B.

SUPPLEMENTARY FILE S6 Details of the two schools of network nodes presented in Supplementary File S5. This file contains 10 tabs: 1) School_A [PG(34_4)]_nodes: PG(34:4) and 371 genes in its immediate neighbourhood, their respective association degree in the entire network and in this subnetwork, and overall association strength; 2) PG(34_4)_edges: connection edges of this subnet; 3) PG(34_4)_GO: Gene ontology enrichment analysis (GOEA) of the 371 genes; 4) School_B_nodes: PG(34:3), PG(36:6) and 105 genes in their immediate neighbourhood, their respective association degree in the entire network and in respective subnetwork; 5) - 10) the nodes, edges and GOEA of the genes in PG(34:3) and PG(36:6) subnets.

SUPPLEMENTARY FILE S7 The network property of the 214 genes in the immediate neighbourhood of three homoeologs of the VRN1 gene (VRN-A1, VRN-B1, VRN-D1). This file contains three tabs: 1) The eight VRN genes; 2) the 217 nodes, 3) the 19570 edges.

SUPPLEMENTARY FILE S8 Gene-GO associations used for gene ontology enrichment analysis in this study. These associations were assemble from EnselblPlants release 51. The gene ID of IWGSC Refseq v1.2 was converted to IWGSC RefSeq v2.1 based on the ID mapping provided by Zhu et al., (2021).

SUPPLEMENTARY FILE S1 The third principal component (PC3) separates winter-habit genotypes from the spring-habit ones in each pair of the NILs when treated with cold. (A) PC3 vs. PC1, (B) PC3 vs. PC2.

SUPPLEMENTARY FILE S2 Frequency distribution of DEGs presented by DEFE patterns of the two pairs of NILs between winter-habit and spring-habit genotypes. (A) C series: cold-treated samples; (B) K series: control samples.

SUPPLEMENTARY FILE S3 Contrast in network association strength: (A) winter-habit genes were generally well associated within the group; (B) less than half of the spring-habit genes were loosely associated another gene in the group.

SUPPLEMENTARY FILE S4 Contrast between cold hardy and anti-hardy genes in network association perspective: (A) cold hardy genes were generally well associated within the group, the four WHGs and PG(34:3) and PG(36:6) are highlighted; (B) only 1/3 of the anti-hardy genes were loosely associated with another gene.

SUPPLEMENTARY TABLE S1 Range of PC scores of the genes up- or down-regulated specifically to winter-habit or spring-habit genotypes.

## Glossary

ABA: abscisic acid
AS: association strength
CBF: C-repeat binding factor
COR: cold regulated gene
COR413-TM1: COLD REGULATED 314 THYLAKOID MEMBRANE 1
DEFE: Differential Expression Feature Extraction
DEG: differentially expressed gene
DGDG: digalactosyldiacylglycerol
DREB1: dehydration-responsive element-binding protein 1
FC: fold change
FLC: FLOWERING LOCUS C
GDSL: amino acid sequence motif consisting of Gly, Asp, Ser, and Leu around the active site Ser
GO: Gene Ontology
IWGSC: International Wheat Genome Sequencing Consortium
M: Manitou
MGDG: monogalactosyldiacylglycerol
MYB: myeloblastosis domain containing transcription factor
N: Norstar
NAM: no apical meristem
NIL: near-isogenic line
PC: principal component: phosphatidylcholine
PC: principal component: phosphatidylcholine
PCA: principal component analysis
PE: phosphatidylethanolamine
PG: Phosphatidylglycerol
PI: phosphatidylinositols
PPT: palmitoyl-protein thioesterase
ROS: reactive oxygen species
SA: salicylic acid
SHG: spring-habit gene
SN: spring Norstar
TF: transcription factor
TOM: topology overlap matrix
VRN: vernalization gene
WGCNA: weighted gene correlation network analysis
WHG: winter-habit gene
WM: winter Manitou
