# Supplementary material for: Computational genomics insights into cold acclimation in wheat
Source: Front Genet. 2022 Oct 20;13:1015673. doi: 10.3389/fgene.2022.1015673 (PMC9632429; doi:10.3389/fgene.2022.1015673)
Supplement: Supplementary file 1 [file DataSheet2.pdf]

Supplementary File 5

Two Schools of Network Nodes Represented by Phosphatidylglycerol Lipids

Phosphatidylglycerol (PG) is a major constitute of the thylakoid membranes. PG(34:4), which has a fatty acid 18:3 at the *sn-1* position and a t16:1 at the *sn-2* position of the glycerol backbone, represents about 80% of all PGs, and was previously found to be negatively associated with development of cold hardiness (Li et al 2021). PG(34:3) and PG(36:6), on the other hand, were positively associated with cold acclimation, as shown in our result, even though they appeared to represent less amount than PG(34:4) among PGs. These drew our interests in genes closely associated with these PGs from a network perspective. We specifically focused on their respective sub-networks.

PG(34:4) is synthesized exclusively within the chloroplast (Zhou et al., 2017) and plays a major role in dynamics of plastid PGs and influence other PGs localized in the plastid (Afitlhile et al., 2021). In the overall network, PG(34:4) had a connection degree of 404 including 371 genes. This subnet of 372 nodes was connected with over 16 thousand edges with an average degree per node of 89.61. The association strength of the network was 0.2409 (**Figure 1**). The top 50 highly enriched GO terms ($p < 5E-04$) among the 371 genes in the subnet included siroheme metabolic process, uroporphyrin-III C-methyltransferase activity, precorrin-2 dehydrogenase activity, sirohydrochlorin ferrochelatase activity, cellular amino acid biosynthetic process, homoserine dehydrogenase activity, carbohydrate metabolic process, chloroplast stroma, cobalamin biosynthetic process, hydrolase activities hydrolyzing O-glycosyl compounds and acting on glycosyl bonds, alpha-amino acid biosynthetic process, C-methyltransferase activity, heme biosynthetic process, metal ion binding, imidazoleglycerol-phosphate synthase activity, positive regulation of short-day photoperiodism and flowering, cation binding, amylase activity, monosaccharide metabolic process, xylulokinase activity, calcium ion binding, carbon-carbon lyase activity, pigment biosynthetic process, oxo-acid-lyase activity, pentose metabolic process, alpha-amylase activity, glutamine family amino acid metabolic process, glutathione hydrolase activity, regulation of flower development, regulation of shoot system development, pentosyltransferase activity, long-day photoperiodism, and porphyrin-containing compound biosynthetic process (**Supplemental File S6** tab **PG(34_4)_GO**).

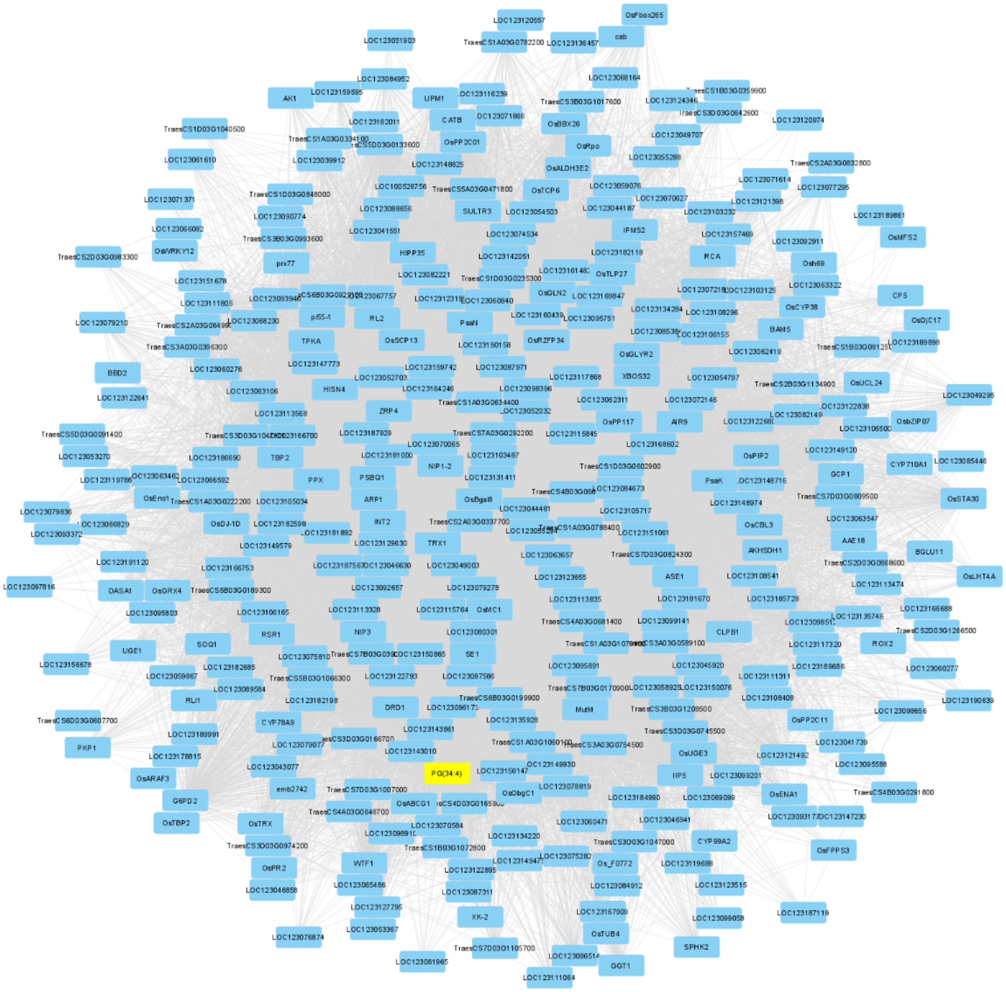

Figure 1. School A subnetwork represented by PG(34:4).

Unlike PG(34:4), at least a portion of the PG(36:6) is synthesized outside of plastids (Zhou et al., 2017, Afitlhile et al., 2021). In this study, the PG(34:3) and PG(36:6) subnets were highly inter-associated with 48 common nodes. The PG(34:3) subnet had 99 nodes with association strength 0.7311 and the PG(36:6) subnet had 55 nodes with association strength 0.6466 (**Figure 2**). Not surprisingly, both PG(34:3) and PG(36:6) subnets shared significant number of enriched GO terms and were enriched with lipid transport and localization, chloroplast inner membrane, oxalate-CoA ligase activity, positive regulation of post-embryonic development, CoA-ligase and acid-thiol ligase activities, cell wall organization or biogenesis, copper and metallo chaperone activities. Specifically, the PG(34:3) subnet was enriched with cellular response to reactive oxygen species including oxygen radical and superoxide, cellular oxidant detoxification, peptidyl-prolyl cis-trans isomerase activity, glutamate-5-semialdehyde dehydrogenase activity.

Whereas, the PG(36:6) subnet was specifically enriched with carbohydrate localization and storage, defense response to fungus, chloroplast envelope (**Supplemental File S6** tabs **PG(34_3)_GO** and **PG(36_6)_GO**).

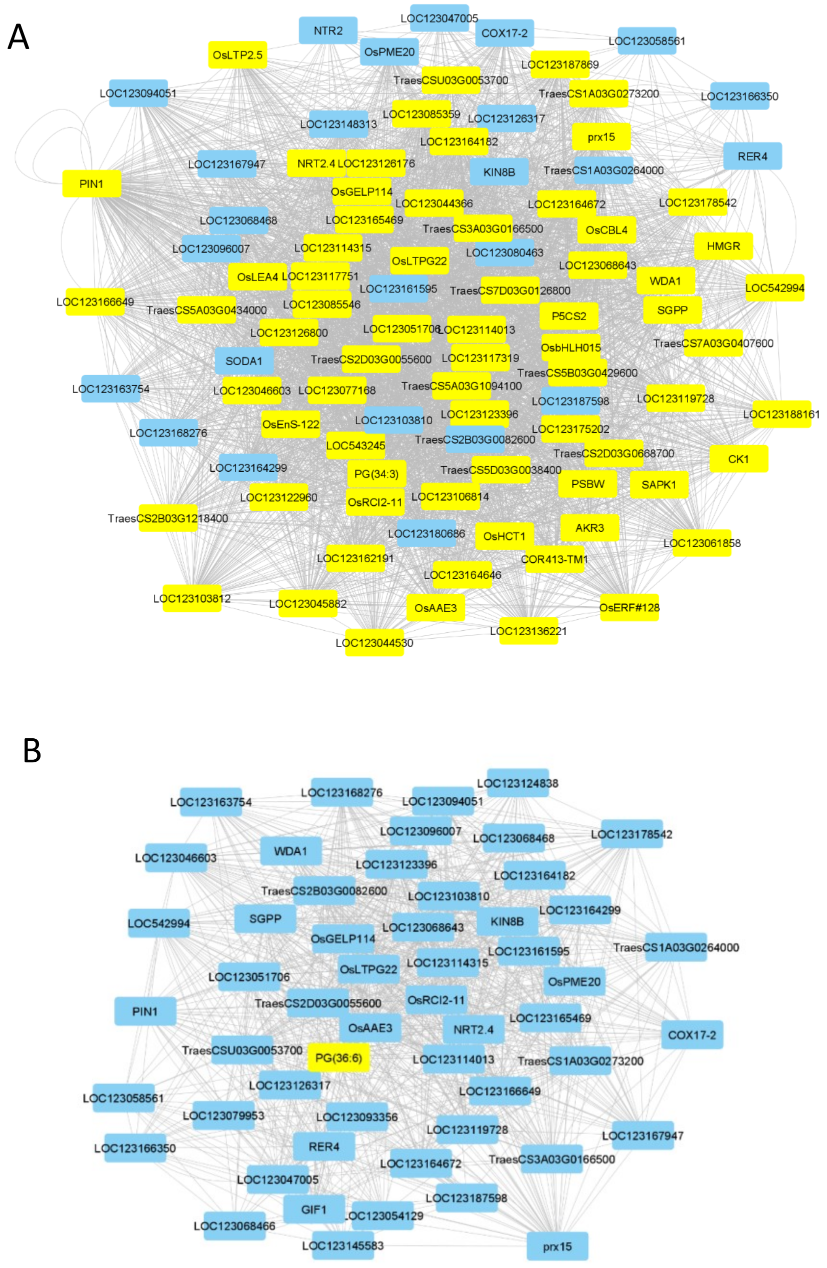

Figure 2. Subnetworks of school B nodes. *a*) phosphatidylglycerol lipid PG(34:3) subnet, nodes directly associated with COR413-TM1 are highlighted. *b*) PG(36:6) subnet.

The results of PG subnetworks provided evidence of two schools of network nodes: PG(34:4) represented school A consisting of all nodes (genes and lipids) that directly associated with this PG. PG(34:3) and PG(36:6) represented school B consisting of all nodes (genes and lipids) directly associated with these two PGs; Network propagation analysis indicated that all nodes within each school were highly integrated; but these two schools were distinctive to each other, without any common neighboring nodes. In addition, as described above, these two groups of PGs contributing to winter-habit genes (WHGs) and cold hardiness in opposite ways. PG(34:4) was negatively correlated with WHGs and cold hardy genes. Whereas, PG(34:3) and PG(36:6) were highly correlated with >50 WHGs and fully correlated with all cold hardy genes. There were 22 cold hardy genes and one WHG and several homoeologs of WHGs in School B. Also, further investigation into PCA characteristics of the membership in the two schools revealed that genes in these two schools were in distinctive niches in PC1 and PC2 two dimensional space (**Figure 3**).

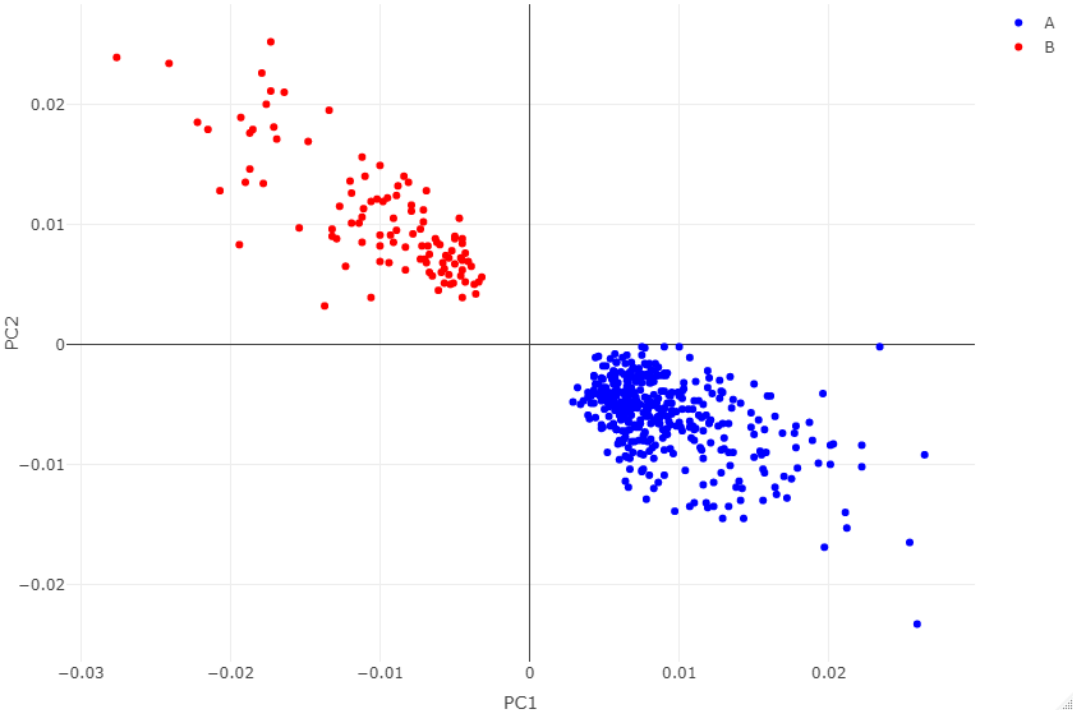

Figure 3. Distribution of genes from the two schools of lipid-gene subnetworks in the two dimensional principal component space. Where, PC1 and PC2, are the principal components 1, and 2. A: nodes in School A [PG(34:4) subnet], B: nodes in School B [PG(34:3) and PG(36:6) subnets]. Details are available in **Supplementary File S6**.

References

Afitlhile, M., Worthington, R., Baldric, J. (2021). The toc132toc120 heterozygote mutant of *Arabidopsis thaliana* accumulates decreased levels of the major chloroplast lipids. Phytochemistry 184:112652. doi: 10.1016/j.phytochem.2020.112652.

Li, Q., Shen, W., Mavraganis, I., Wang, L., Gao, P., Gao, J., et a. (2021). Elucidating the biochemical basis of trans-16:1 fatty acid change in leaves during cold acclimation in wheat. Plant-Environment Interactions 2:101-111. doi: 10.1002/pei3.10044.

Zhou, Y., Hölzl, G., Vom Dorp, K., Peisker, H., Melzer, M., Frentzen, M., Dörmann, P. (2017). Identification and characterization of a plastidial phosphatidylglycerophosphate phosphatase in *Arabidopsis thaliana*. Plant J. 89: 221-234. doi: 10.1111/tpj.13378.
