# Supplementary material for: Computational genomics insights into cold acclimation in wheat
Source: Front Genet. 2022 Oct 20;13:1015673. doi: 10.3389/fgene.2022.1015673 (PMC9632429; doi:10.3389/fgene.2022.1015673)
Supplement: Supplementary file 5 [file DataSheet1.PDF]

Supplementary Figures & Tables

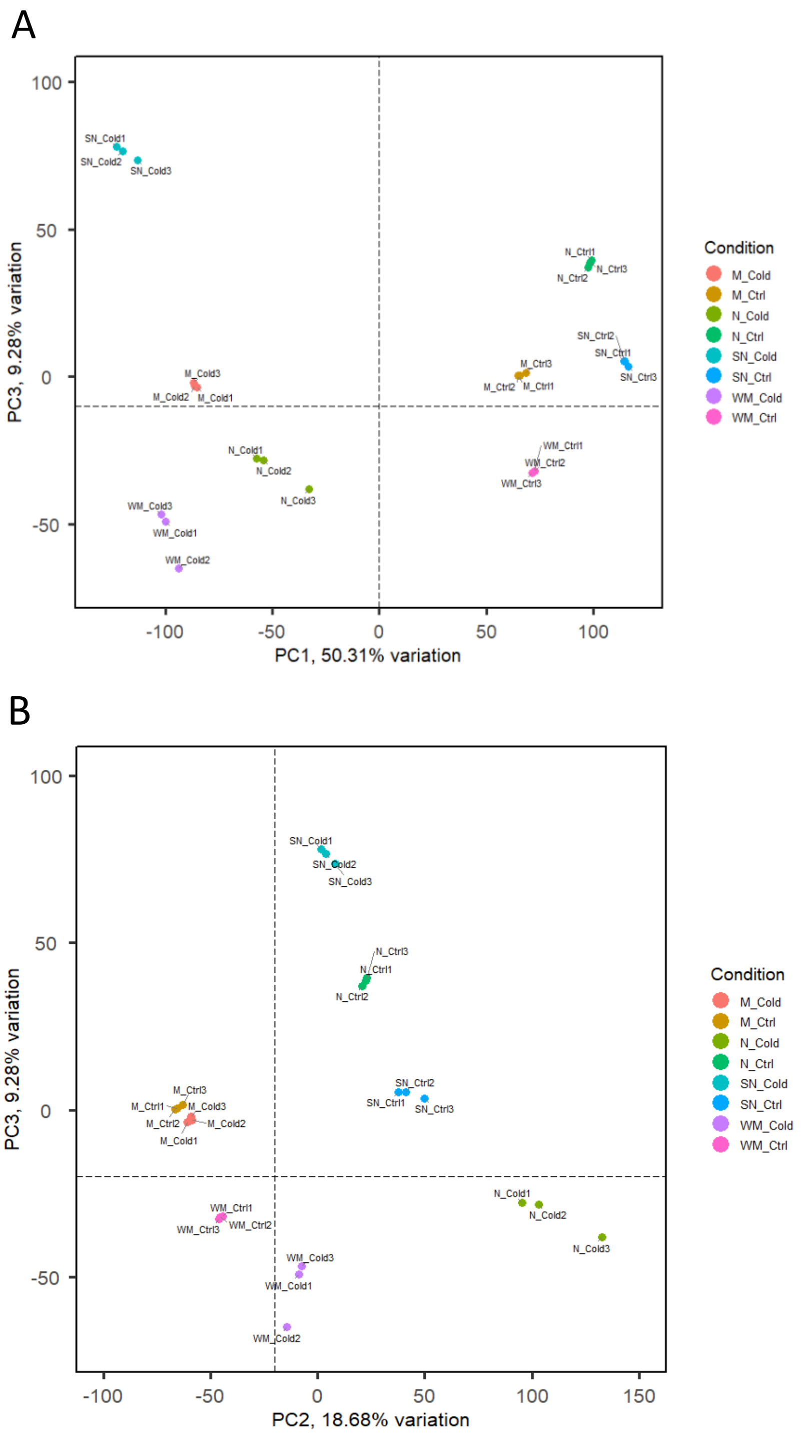

Supplementary Figure S1. The third principal component (PC3) separates winter-habit genotypes from the spring-habit ones in each pair of the NILs when treated with cold. (A) PC3 vs PC1, (B) PC3 vs PC2.

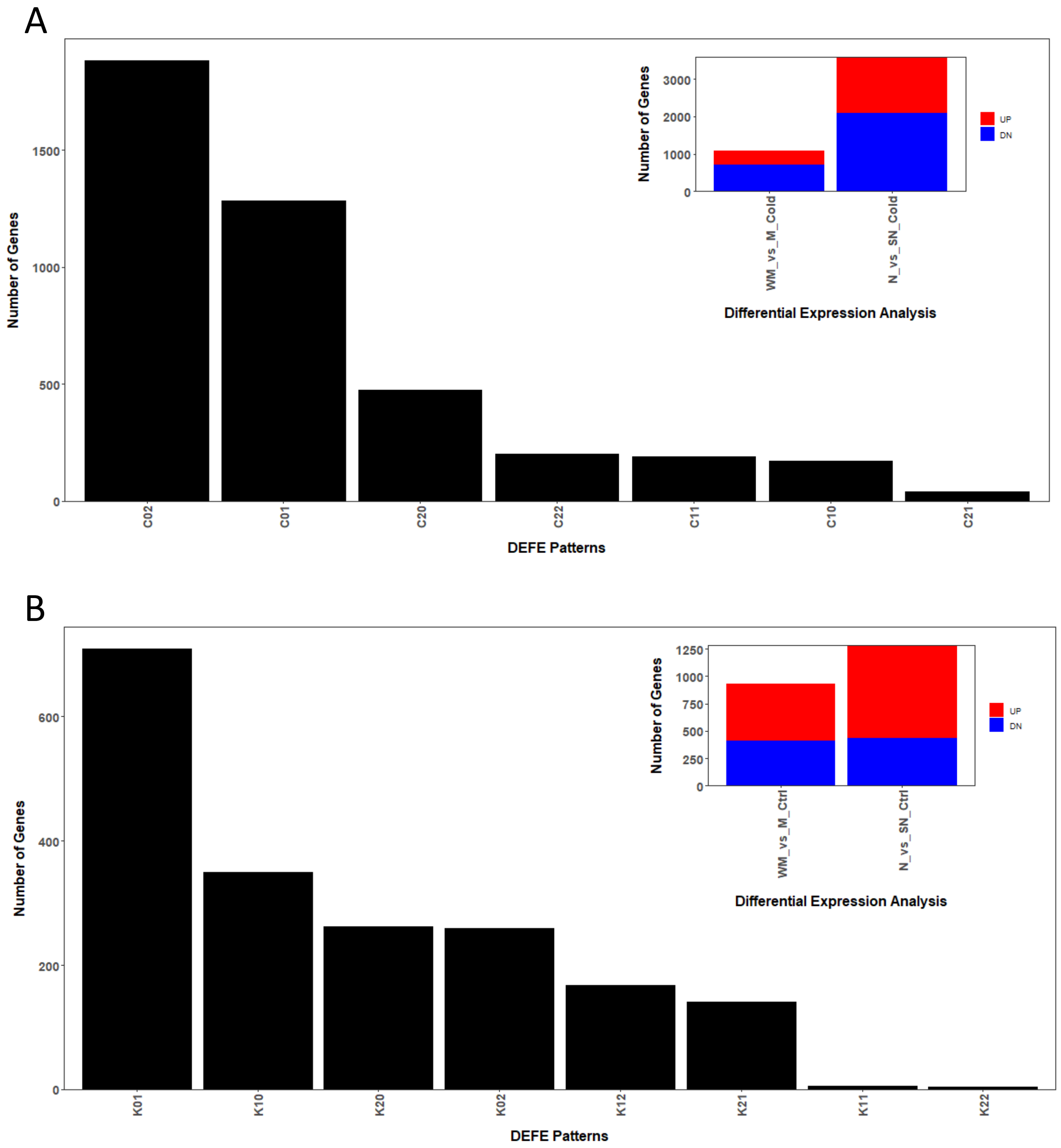

Supplementary Figure S2. Frequency distribution of DEGs presented by DEFE patterns of the two pairs of NILs between winter-habit and spring-habit genotypes. (A) C series: cold-treated samples; (B) K series: control samples.

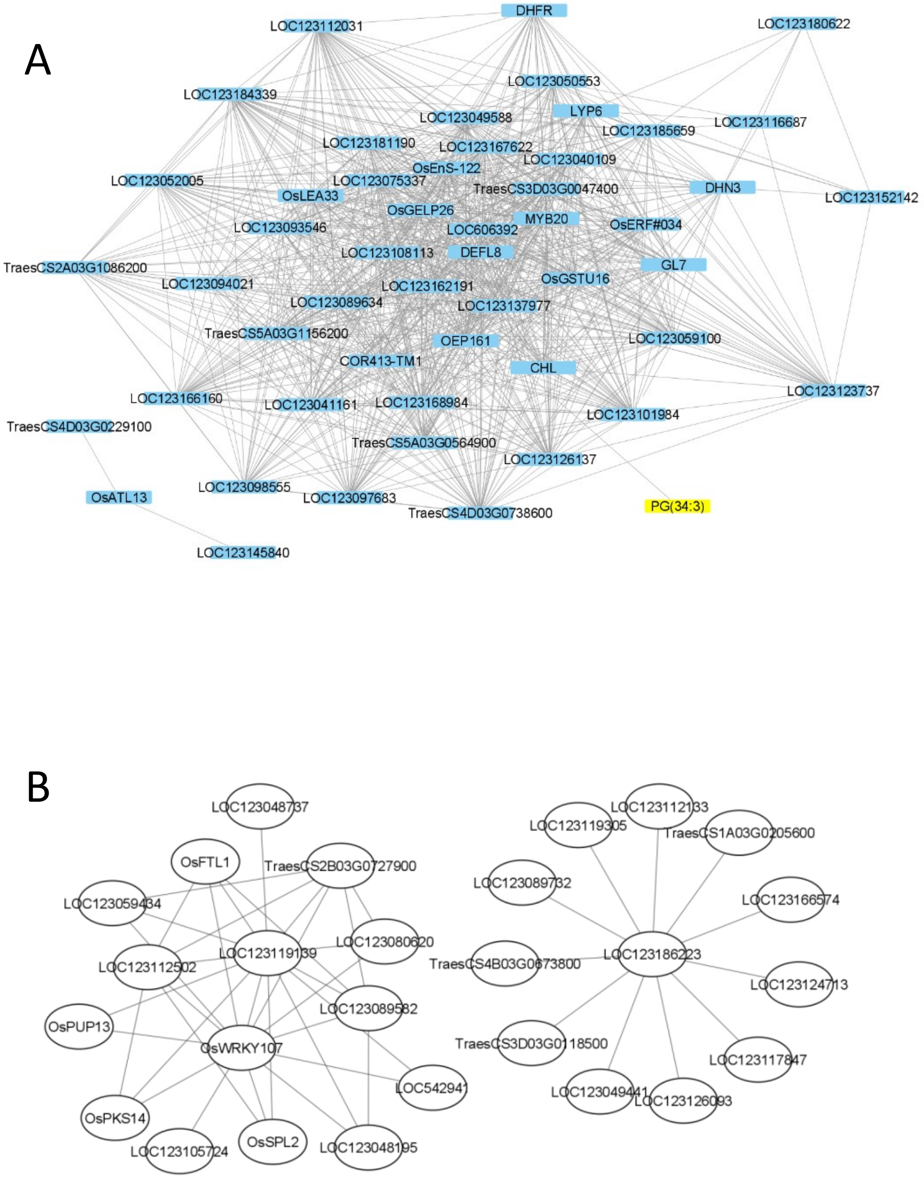

Supplementary Figure S3. Contrast in network association strength: (A) winter-habit genes were generally well associated within the group; (B) less than half of the spring-habit genes were loosely associated with another gene in the group.

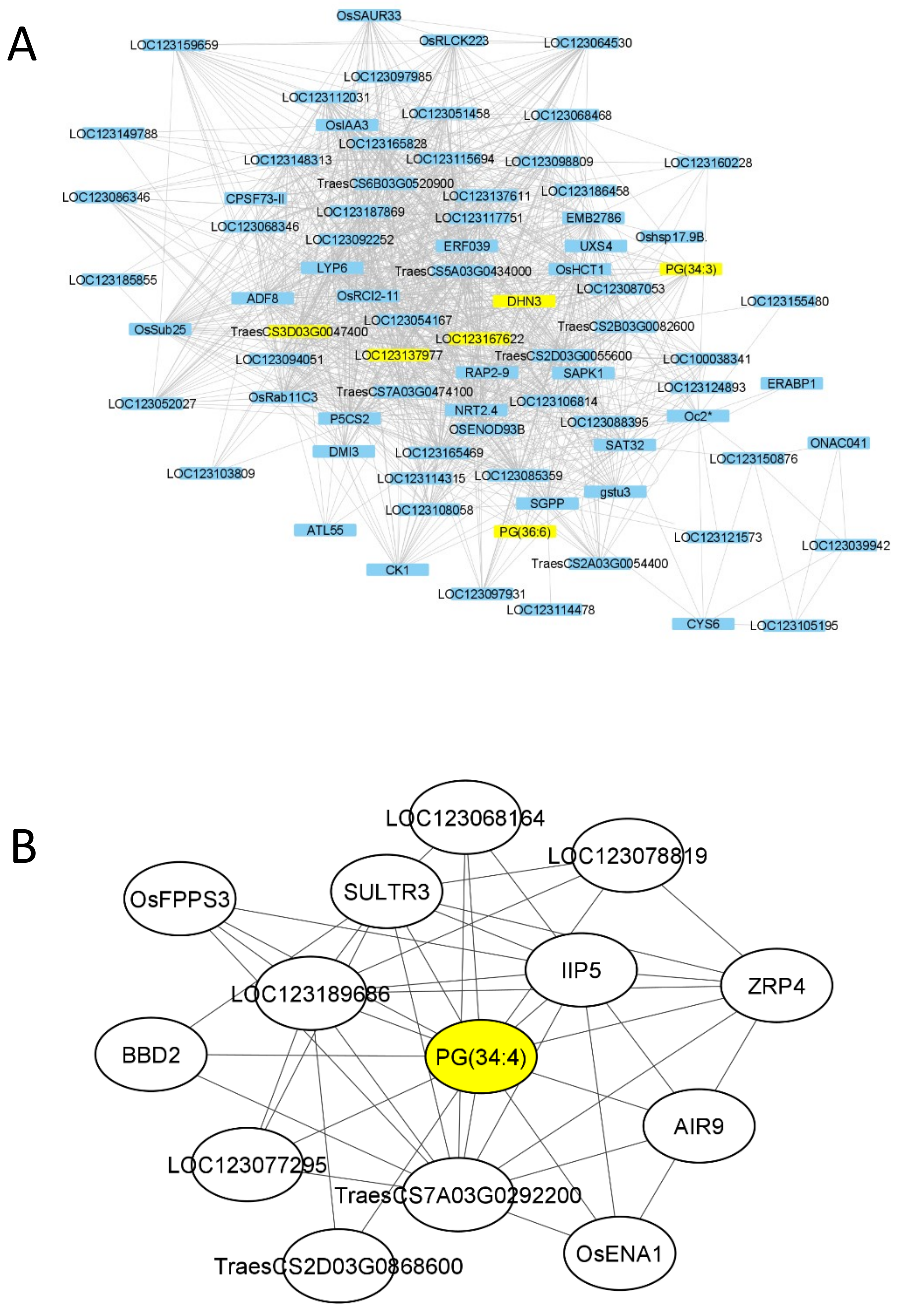

Supplementary Figure S4. Contrast between cold hardy and anti-hardy genes in network association perspective: (A) cold hardy genes were generally well associated within the group, the four winter-habit genes and PG(34:3) and PG(36:6) are highlighted; (B) only 1/3 of the anti-hardy genes were loosely associated with another gene.

Supplementary Table S1. Range of PC scores of the genes up- or down-regulated by specific to winter-habit or spring-habit genotypes

		PC1	PC2	PC3
Up in winter-habit	Max	-0.0020	0.0335	-0.0026
P0101∩C11∩K00	Min	-0.0143	0.0024	-0.0437
Up in spring-habit	Max	-0.0053	0.0001	0.0325
P1010∩C22∩K00	Min	-0.0201	-0.0338	-0.0072
Down in winter-habit	Max	0.0128	-0.0093	0.0264
P0202∩C22∩K00	Min	0.0021	-0.0192	0.0035
Down in spring-habit	Max	0.0135	0.0094	-0.0039
P2020∩C11∩K00	Min	0.0069	0.0091	-0.0087
